# SRSF1 acts as an IFN-I-regulated cellular dependency factor decisively affecting HIV-1 post-integration steps

**DOI:** 10.3389/fimmu.2022.935800

**Published:** 2022-11-15

**Authors:** Helene Sertznig, Fabian Roesmann, Alexander Wilhelm, Delia Heininger, Barbara Bleekmann, Carina Elsner, Mario Santiago, Jonas Schuhenn, Zehra Karakoese, Yvonne Benatzy, Ryan Snodgrass, Stefan Esser, Kathrin Sutter, Ulf Dittmer, Marek Widera

**Affiliations:** ^1^ Institute for Virology, University Hospital Essen, University Duisburg-Essen, Essen, Germany; ^2^ Institute for Medical Virology, University Hospital Frankfurt, Goethe University Frankfurt am Main, Frankfurt am Main, Germany; ^3^ Department of Medicine, University of Colorado Denver, Aurora, CO, United States; ^4^ Institute of Biochemistry I, Faculty of Medicine, Goethe-University Frankfurt am Main, Frankfurt, Germany; ^5^ Clinic of Dermatology, University Hospital, University of Duisburg-Essen, Essen, Germany

**Keywords:** HIV-1, interferon, ISG (interferon stimulated genes), repressed genes, transcription, alternative splicing, SRSF1, SF2/ASF

## Abstract

Efficient HIV-1 replication depends on balanced levels of host cell components including cellular splicing factors as the family of serine/arginine-rich splicing factors (SRSF, 1–10). Type I interferons (IFN-I) play a crucial role in the innate immunity against HIV-1 by inducing the expression of IFN-stimulated genes (ISGs) including potent host restriction factors. The less well known IFN-repressed genes (IRepGs) might additionally affect viral replication by downregulating host dependency factors that are essential for the viral life cycle; however, so far, the knowledge about IRepGs involved in HIV-1 infection is very limited. In this work, we could demonstrate that HIV-1 infection and the associated ISG induction correlated with low SRSF1 levels in intestinal lamina propria mononuclear cells (LPMCs) and peripheral blood mononuclear cells (PBMCs) during acute and chronic HIV-1 infection. In HIV-1-susceptible cell lines as well as primary monocyte-derived macrophages (MDMs), expression levels of SRSF1 were transiently repressed upon treatment with specific IFNα subtypes in vitro. Mechanically, 4sU labeling of newly transcribed mRNAs revealed that IFN-mediated SRSF1 repression is regulated on early RNA level. SRSF1 knockdown led to an increase in total viral RNA levels, but the relative proportion of the HIV-1 viral infectivity factor (Vif) coding transcripts, which is essential to counteract APOBEC3G-mediated host restriction, was significantly reduced. In the presence of high APOBEC3G levels, however, increased LTR activity upon SRSF1 knockdown facilitated the overall replication, despite decreased vif mRNA levels. In contrast, SRSF1 overexpression significantly impaired HIV-1 post-integration steps including LTR transcription, alternative splice site usage, and virus particle production. Since balanced SRSF1 levels are crucial for efficient viral replication, our data highlight the so far undescribed role of SRSF1 acting as an IFN-modulated cellular dependency factor decisively regulating HIV-1 post-integration steps.

## Introduction

Type I interferons (IFN-I), which, among others, include 12 individual IFNα subtypes and IFNβ, play a crucial role in the early innate immune defense against viral infections including HIV-1 (1, 2). After viral sensing *via* pattern recognition receptors (PRRs) like the Toll-like receptors (TLRs) or the cytosolic DNA sensor cyclic GMP-AMP synthase (cGAS), synthesis and secretion of IFN-I are induced (3–5). Binding of extracellular IFN-I to the IFNα/β-receptor (IFNAR) induces signaling *via* the JAK/STAT-pathway and leads to the transcription of hundreds of IFN-stimulated genes (ISGs), such as host restriction factors or transcription factors establishing an antiviral state within the host and bystander cells (6–8). Among others, potent members of ISGs with anti-retroviral activity include ISG15 (IFN-stimulated gene 15) (9–11), APOBEC3G (apolipoprotein B mRNA editing enzyme and catalytic polypeptide-like 3G) (12, 13), tetherin (14, 15), Mx-2 (Myxovirus resistance-2) (16, 17), SAMHD1 (SAM domain and HD domain-containing protein 1 (18–20), and IFITM1-3 (Interferon-induced transmembrane protein 1-3) (21, 22). Despite a high sequence homology between IFNα subtypes and binding to the same receptor (IFNAR), all 12 individual IFNα subtypes were shown to exert different antiviral activities such as the induction of a distinct set of ISGs (23–25). While several IFNα subtypes were shown to elicit antiviral activity against human immunodeficiency virus type 1 (HIV-1), IFNα14 has proven to be the most potent subtype (26, 27). However, IFNα2, which is the sole subtype currently used in clinical treatments such as hepatitis B virus (HBV) infection (28), showed only limited antiviral activity against HIV-1.

In addition to the well-described induction of ISGs and other immunomodulatory functions, IFN-I also induces the repression of specific genes, termed IFN-repressed genes (IRepG) (29, 30). Several transcription factors were identified as IRepGs, suggesting a role in the restriction of cellular and viral gene expression. Furthermore, distinct cellular factors including RNA-binding proteins (RBPs) that are crucial for viral replication could be identified as IRepGs (29, 31). Thus, their downregulation might represent a possible cellular defense mechanism limiting essential host dependency factors for HIV-1 replication.

Once integrated into the host cell genome, HIV-1 exploits the cellular transcription and RNA processing apparatus for viral gene expression (32, 33). The proviral DNA, which is under the transcriptional control of the LTR promoter, is transcribed as a full-length precursor mRNA (pre-mRNA) and undergoes excessive alternative splicing to extract the full genetic content of the short and compact genome (34–37). The usage of at least five splice donor sites (SD1 to SD4 including the alternative splice site SD2b) and multiple splice acceptor sites (SA1 to SA7) in various combinations allows the production of balanced levels of essential viral mRNAs. The recognition of these splice sites is regulated not only by their intrinsic strength but also by a complex network of splicing regulatory elements (SREs) located on the viral pre-mRNA (38, 39), both together referred to as the “splicing code” (40). Interaction of specific RBPs with SREs can, in a position-dependent manner, inhibit or promote the recognition and usage of a specific splice site (35, 37, 41, 42). These cellular splicing factors were defined as host dependency factors, which are essential for HIV-1 replication, but not lethal to the host cell upon gene silencing (43–47).

Serine/arginine-rich splicing factors (SRSFs) represent a large family of RBPs (48–51). Two main structural features are conserved among all SRSF proteins, the protein-interacting RS domain, which is rich in arginine and serine (RS) dipeptides, and the RNA recognition motif (RRM), which enables interactions with pre-mRNAs. SRSF proteins are generally characterized by their ability to interact with both RNA and protein structures simultaneously (52–54). SRSF protein activity and subcellular localization are mediated *via* post-translational modifications, such as phosphorylation (55), acetylation (56), or methylation (57–59). Several members of this protein family have been shown to be crucial regulators of alternative splice site usage. While SRSF proteins promote binding of U1 snRNP or U2 snRNP to a 5’- or 3’-splice site and thus splice site recognition when binding to an SRE located in an exonic position (60–63), they repress splice site recognition when binding an SRE located in an intronic position, possibly due to steric hindrance (64–66). Furthermore, SRSF proteins were shown to regulate several other steps of gene expression, such as transcription (67, 68), nuclear mRNA export (69, 70), mRNA decay, or translation (71, 72). As the founding member of the SRSF protein family, serine/arginine-rich splicing factor 1 (SRSF1), formerly known as SRp30a or ASF/SF2 (73), was originally identified to promote spliceosomal assembly and pre-mRNA splicing in HeLa cells (74), as well as to regulate alternative splicing of the SV40 pre-mRNA in HEK293 cells (75). SRSF1 contains two RRMs, providing the RNA-binding specificity, and a short RS domain (54). *In vivo* mapping identified the purine-rich sequence GGAGA as consensus motif of the SRSF1 binding site (76, 77).

SRSF1 has been described to play a crucial role in the regulation of gene expression and RNA processing of HIV-1 (78–81). Several SREs within the HIV-1 pre-mRNA are known targets of SRSF1, such as exonic splicing enhancer (ESE) M1/M2 (79), GAR-ESE (78), or ESE3 (80). Binding of SRSF1 to these *cis*-regulatory elements was shown to facilitate the recognition and usage of proximal splice sites. Furthermore, SRSF1 was shown to compete with Tat for a binding site within the TAR region on the LTR promoter, thereby interfering with the Tat-mediated HIV-1 LTR transactivation (82, 83). While high SRSF1 level resulted in enhanced Vpr, but reduced Tat1, Gag, and Env levels (84–86), low levels facilitated the expression of all viral mRNAs indicating a major impact on LTR transcription (84). Thus, SRSF1 represents a host dependency factor and key regulator for efficient HIV-1 RNA processing, enabling the emergence of the protein diversity necessary for efficient viral replication.

In this manuscript, we investigated whether the gene expression levels of SRSFs are influenced through HIV-1 infection and concomitant IFN-I stimulation. We observed a negative correlation between *ISG15* and *SRSF1* mRNA expression, particularly during acute HIV-1 infection. Furthermore, we identified *SRSF1* as a temporary IFN-regulated gene. Our findings suggest that balanced levels of SRSF1 are crucial for efficient HIV-1 replication, as in particular overexpression and knockdown resulted in severe impairments of HIV-1 post-integration steps at the level of LTR transcription, alternative splicing, or virus production. This work highlights the so far undescribed role of SRSF1 as IFN-regulated cellular effector molecule, decisively affecting HIV-1 LTR transcription and RNA processing, thereby contributing to the IFN-induced unfavorable cellular environment for HIV-1 replication.

## Results

### SRSF transcript levels are significantly lower in HIV-1-infected individuals

**Figure 1:**
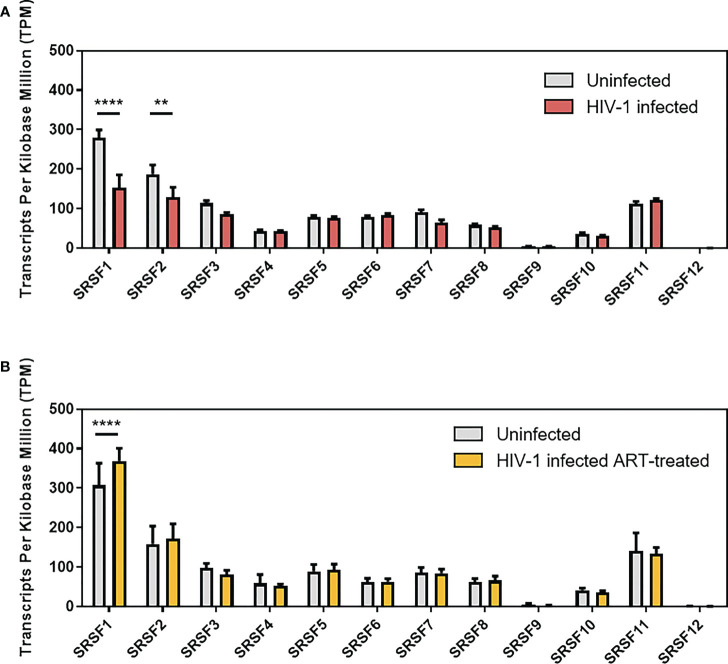
Gene expression levels of *SRSFs* in treatment-naïve or ART-treated HIV-1-infected individuals. Transcript levels of SRSF genes were measured in lamina propria mononuclear cells (LPMCs) from the gut using RNA-sequencing analysis (**Supplementary Table 1**). Comparison of transcript levels from **(A)** treatment-naïve HIV-1-infected and healthy individuals and **(B)** ART-treated HIV-1-infected and healthy individuals. TPM are depicted as mean (+ SD) for **(A)** 19 HIV-1-infected and 13 uninfected individuals and **(B)** 14 ART-treated and 11 uninfected individuals. Groups were compared with two-way ANOVA with Bonferroni *post-hoc* test (**p < 0.01, and ****p < 0.0001).

**Figure 2:**
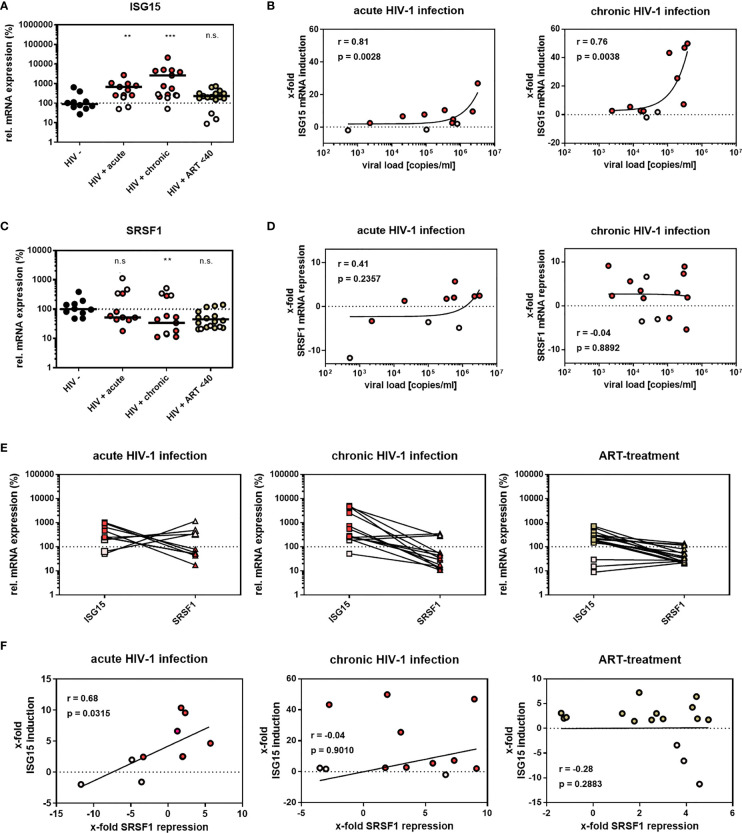
*SRSF1* and *ISG15* expression levels inversely correlate upon HIV-1 infection. **(A, C)** RT-qPCR determined the relative mRNA expression levels of **(A)** *ISG15* and **(C)** *SRSF1* in PBMCs from acutely and chronically HIV-1-infected patients, either naïve or under ART treatment as well as healthy donors. ACTB was used for normalization. Kruskal–Wallis test with the Dunn’s *post-hoc* multiple comparisons test was used to determine whether the difference between the group of samples reached the level of statistical significance (**p < 0.01, ***p < 0.001 and ns, not significant). **(B, D)** Correlation between plasma viral load of HIV-1-infected individuals and *ISG15* and *SRSF1* mRNA expression. RT-qPCR analysis was performed to determine *ISG15* and *SRSF1* mRNA expression. Pearson correlation was calculated between plasma viral load and **(B)** *ISG15* or **(D)** *SRSF1* expression for acutely and chronically HIV-1-infected patients. Pearson correlation coefficient (*r*) and *p*-value (*p*) are indicated. **(E)** Comparison of relative *ISG15* and *SRSF1* mRNA levels for individual patients. **(F)** Calculated correlation between *x*-fold repression of *SRSF1* mRNA levels and *x*-fold induction of *ISG15* mRNA levels for all patient groups. Pearson correlation coefficient (*r*) and *p*-value (*p*) are indicated. Data points from healthy donors were depicted in black, while data points from treatment-naïve HIV-1-infected individuals were shown in red. ART-treated patients are colored in green. Patients with no or low *ISG15* mRNA induction upon HIV-1 infection were considered as low responders without IFN signature and were thus excluded from statistical analysis (light gray). This patient cohort included 10 uninfected donors, 8 acutely HIV-1-infected patients, 11 chronically HIV-1-infected patients, and 13 HIV-1-infected patients under ART treatment.

In a previous RNA-sequencing based study, we were able to demonstrate that compared to healthy donors, transcript levels of specific host restriction factors, such as tetherin, Mx-2, or APOBEC3G, were upregulated in intestinal lamina propria mononuclear cells (LPMCs) of HIV-1-infected treatment-naïve or antiretroviral therapy (ART)-treated individuals (87). To analyze whether host dependency factors including RBPs might also be altered upon HIV-1 infection, RNA-sequencing data were reanalyzed focusing on the gene expression of cellular splicing factors including *SRSF* family members. Significantly lower levels of mRNA transcripts were detected for *SRSF1* (1.8-fold) and *SRSF2* (1.4-fold) in HIV-1-infected treatment-naïve patients when compared to healthy individuals (**Figure 1A**; **Supplementary Table 1**). Furthermore, *SRSF3* (1.3-fold), *SRSF7* (1.4-fold), *SRSF8* (1.1-fold), and *SRSF10* (1.2-fold) transcript levels were also lower in this cohort, albeit without statistical significance (**Figure 1A**). In the cohort of HIV-1-infected and ART-treated patients, mRNA transcript levels of *SRSF2*, *SRSF7*, and *SRSF8* were comparable to the levels observed in the healthy control group, while *SRSF3* (1.2-fold) and *SRSF10* (1.1-fold) mRNA expression levels were still lower (**Figure 1B**). Of particular interest, transcript levels of *SRSF1* were significantly higher (1.2-fold) in HIV-1-infected ART-treated patients when compared to uninfected donors (**Figure 1B**). No significant difference in transcript levels were observed upon HIV-1 infection, either treatment-naïve or under ART treatment, for *SRSF4*, *SRSF5*, *SRSF6*, and *SRSF11* when compared to healthy individuals (**Figures 1A, B**). In all patient groups, *SRSF9* and *SRSF12* transcript levels were only marginally above the limit of detection (**Figures 1A, B**). Since, concomitant to the elevated ISG profile (87), mRNA transcript levels of several *SRSFs* were lower in HIV-1-infected individuals, a possible correlation with interferon signaling and chronic inflammation was further investigated. Since SRSF1 was the most significant differentially expressed gene of the SRSF family in LPMCs of HIV-1-positive patients and was also previously described to be crucially involved in HIV-1 post-integration steps (78–81), the expression profile of SRSF1 was analyzed in peripheral blood mononuclear cells (PBMCs) of another cohort of HIV-1-infected individuals at various infection stages (88). For this purpose, PBMCs were isolated from HIV-1-positive individuals during acute infection (Fiebig I–V), chronic infection (Fiebig VI), or chronic infection phase under ART treatment as well as from HIV-1-negative healthy donors. Total cellular RNA was isolated and subjected to RT-qPCR analysis. To evaluate whether the patient cohort was representative, *ISG15* mRNA expression was determined as a surrogate marker for IFN signature, since it was previously demonstrated that ISG expression levels are induced upon HIV-1 infection in PBMCs (23). When compared to healthy individuals, *ISG15* mRNA expression levels were 5.6- and 7.3-fold higher in acutely and chronically HIV-1-infected patients, respectively, while ART-treated patients only had 2.2-fold higher *ISG15* mRNA levels (**Figure 2A**). The virus-induced IFN signature was found to be proportional to the plasma viral load upon both acute and chronic HIV-1 infection (**Figure 2B**). Next, we performed a specific RT-qPCR analysis to investigate whether the *SRSF1* repression might correlate with ISG induction in HIV-1-infected patients. For acutely and chronically HIV-1-infected patients, 2- and 2.6-fold lower levels of *SRSF1* mRNA, respectively, were detected in contrast to healthy donors (**Figure 2C**). Upon ART treatment, *SRSF1* mRNA expression levels were 2.6-fold lower when compared to uninfected individuals (**Figure 2C**). While *SRSF1* mRNA levels were lower in the majority of the samples derived from acutely and chronically HIV-1-infected treatment-naïve patients in contrast to the healthy control group, *SRSF1* expression levels were increased in some patients (**Figure 2C**). Since most individuals with higher levels of *SRSF1* mRNA also had no or only marginally induced levels of *ISG15* mRNA, they were defined as low responders and excluded from statistical analysis (marked in light pink). No significant correlation was detected between the repression of *SRSF1* mRNA and the plasma viral load of acutely and chronically HIV-1-infected patients (**Figure 2D**). Generally, high levels of *ISG15* mRNA expression were concomitant with a strong repression of *SRSF1* mRNA levels in single individuals (**Figure 2E**). The determination of Pearson correlation coefficients revealed a negative correlation between *ISG15* and *SRSF1* mRNA expression upon acute HIV-1 infection (**Figure 2F**). No direct correlation between ISG induction and *SRSF1* repression could be found for the group of chronically HIV-1-infected individuals, which generally represent a heterogeneous cohort due to unmatched infection phases, comorbidities, and other factors (89, 90). Consistent with the previous data, we did not observe any correlation between *ISG15* and *SRSF1* expression in the group of ART-treated individuals (**Figure 2F**).

In conclusion, since we revealed a possible link between the stimulation of ISGs and SRSF1 repression, *SRSF1* might potentially represent an IRepG as part of the immune response to HIV-1.

### The degree of SRSF1 repression is IFNα subtype dependent

We previously showed that IFNα subtypes exert distinct antiviral activities upon HIV-1 infection and that their antiviral potential correlates with the induction of ISGs including host restriction factors (26, 27). To assess whether the observed correlation between ISG induction and *SRSF1* repression in the early immune response upon HIV-1 infection was induced by IFN signaling, the effect of IFNα subtypes (α1, α2, α4, α5, α6, α7, α10, α14, α16, α17, and α21) on the mRNA expression levels of ISG15 and SRSF1 was analyzed.

**Figure 3:**
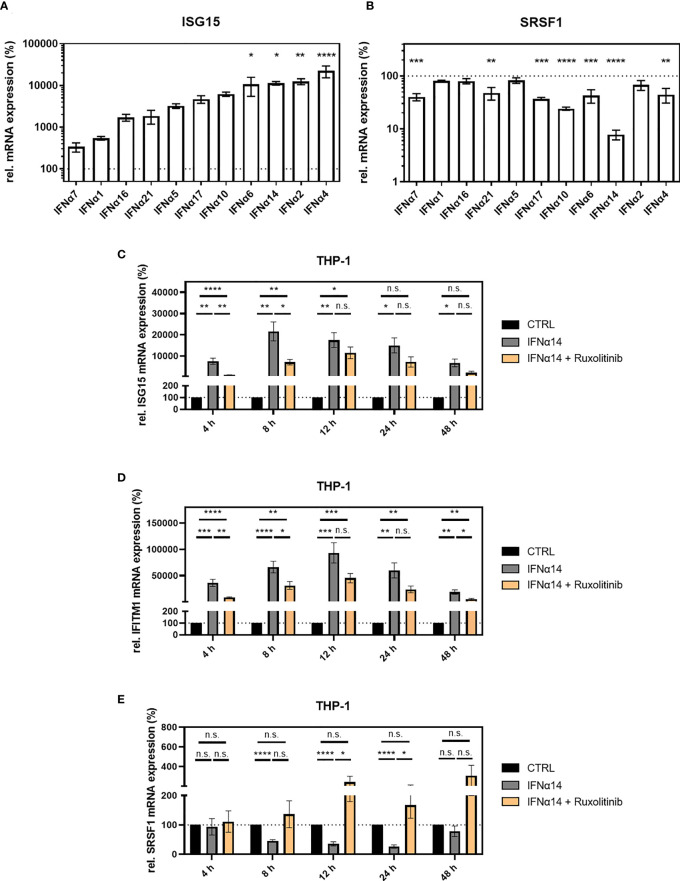
*SRSF1* expression upon stimulation with IFNα subtypes. Differentiated THP-1 cells were treated with the indicated IFN subtype (10 ng/ml). Twenty-four hours post-treatment, cells were harvested, and RNA was isolated and subjected to RT-qPCR for measurement of relative **(A)** *ISG15* and **(B)** *SRSF1* mRNA expression levels. Statistical significance was analyzed performing one-way ANOVA with Dunnett *post-hoc* test (*p < 0.05, **p < 0.01, ***p < 0.001, and ****p < 0.0001). Mean ( ± SEM) of *n* = 3 biological replicates is depicted. **(C–E)** Differentiated THP-1 cells were treated with 1 µM Ruxolitinib or DMSO as mock control1 h before infection using NL4-3 AD8 (1 MOI). Sixteen hours post-infection, cells were washed with PBS and treated with media containing PBS or IFNα14, and either Ruxolitinib or DMSO. At the indicated time points, wells were rinsed with PBS, and cells were subjected to RNA isolation and RT-qPCR analysis to monitor mRNA expression levels of **(C)** ISG15, **(D)** IFITM1, and **(E)** SRSF1. Groups were compared with two-way repeated-measures ANOVA with Tukey’s *post-hoc* test. ns is not significant.

Using a reporter cell line (RPE-ISRE luc) that harbors the firefly luciferase gene under the control of the IFN-inducible ISRE promoter, we evaluated the biological activity of the IFNα subtypes confirming a strong activity except for IFNα1, IFNα7, and IFNα21 (**Supplementary Figure 1**). Next, THP-1 monocytic cells were differentiated into macrophage-like cells using phorbol 12-myristate 13-acetate (PMA) and treated with IFNα subtypes. In general, the intensity of *ISG15* induction reflected the intensity of the luminescent signal in the reporter cells (**Figure 3A** and **Supplementary Figure 1**). Subtypes IFNα2 (124.7-fold), IFNα4 (223.4-fold), IFNα6 (106.2-fold), and IFNα14 (94.5-fold) induced the strongest *ISG15* mRNA expression when compared to the unstimulated control (**Figure 3A**). Treatment with IFNα5 (32.3-fold), IFNα10 (62.1-fold), IFNα16 (17.1-fold), IFNα17 (47.0-fold), and IFNα21 (18.5-fold) led to a moderate but not significant *ISG15* increase, while treatment with IFNα1 (5.4-fold) and IFNα7 (3.4-fold) did not significantly alter *ISG15* mRNA expression (**Figure 3A**). These data were in accordance with previous findings, which described the induction of other ISGs such as Mx2, TRIM22, and APOBEC3G (**Supplementary Figure 2**) upon stimulation with specific IFNα subtypes (23).

The strongest repression in *SRSF1* mRNA expression was observed for subtypes IFNα10 (4.2-fold) and IFNα14 (12.5-fold), while subtypes IFNα4 (2.3-fold), IFNα6 (2.3-fold), IFNα7 (2.5-fold), IFNα17 (2.7-fold), and IFNα21 (2.1-fold) induced a moderate but significant *SRSF1* mRNA repression (**Figure 3B**). Changes in *SRSF1* mRNA expression observed upon stimulation with subtypes IFNα1 (1.3-fold), IFNα2 (1.5-fold), IFNα5 (1.2-fold), and IFNα16 (1.3-fold) were not significant (**Figure 3B**). Remarkably, not all subtypes induced an inverse correlation between *ISG15* induction and *SRSF1* repression. A strong induction in *ISG15* mRNA expression was observed upon treatment with IFNα2, IFNα4, and IFNα6; however, these subtypes only induced a weak repression in *SRSF1* mRNA. IFNα14 induced a disproportionately strong repression in *SRSF1* mRNA levels compared to the stimulation of *ISG15* mRNA expression.

In conclusion, in agreement with previous work (7, 25, 91, 92), the degree of ISG induction was IFNα subtype dependent, suggesting distinct antiviral activities. Generally, all IFNα subtypes induced a repression of *SRSF1* mRNA levels, albeit to a highly varying extent, with IFNα10 and IFNα14 being the most potent. These results substantiate the assumption of *SRSF1* representing an IRepG.

In our further studies, we included the subtypes IFNα2 and IFNα14 as IFNα14 was shown to be the most potent subtype against HIV-1 (27) and induced the strongest downregulation of *SRSF1* mRNA levels (**Figure 3B**), while IFNα2 is the sole IFNα subtype currently in clinical use for the treatment of other viral infections such as HBV (28).

To assess whether the downregulation of SRSF1 is a direct effect of IFN treatment, we added Ruxolitinib, which is a Janus kinase inhibitor (JAK1 and JAK2) and thus blocks the IFN signaling pathway (93). At 4 and 8 h post-treatment, the addition of Ruxolitinib resulted in significantly reduced *ISG15* expression, when compared to unblocked administration of IFNα14 (**Figure 3C**).

Since ISG15 was described to additionally be modulated by a non-Jak1/2 dependent pathway, we monitored the expression of IFITM1 as an additional Jak1/2-dependent ISG. In agreement with previous studies (94). Ruxolitinib significantly reduced *IFITM1* mRNA expression at 4 to 48 h post-treatment, albeit only statistically significant at 4, 8, and 48 h (**Figure 3D**). However, complete inhibition of both ISGs could not be observed, potentially due to JAK1/2-independent pathways. This observation was in agreement with previous studies showing a strong ISG15 induction by viral infections (9). *SRSF1* expression was significantly higher upon treatment with Ruxolitinib after 12 and 24 h of IFNα14 treatment (**Figure 3E**) while no reduction of *SRSF1* mRNA expression was observed in the presence of Ruxolitinib. These results confirm the correlation in the induction of IFN-stimulated genes and SRSF1 repression.

### SRSF1 is an IFN-regulated gene in HIV-1 target cells

**Figure 4:**
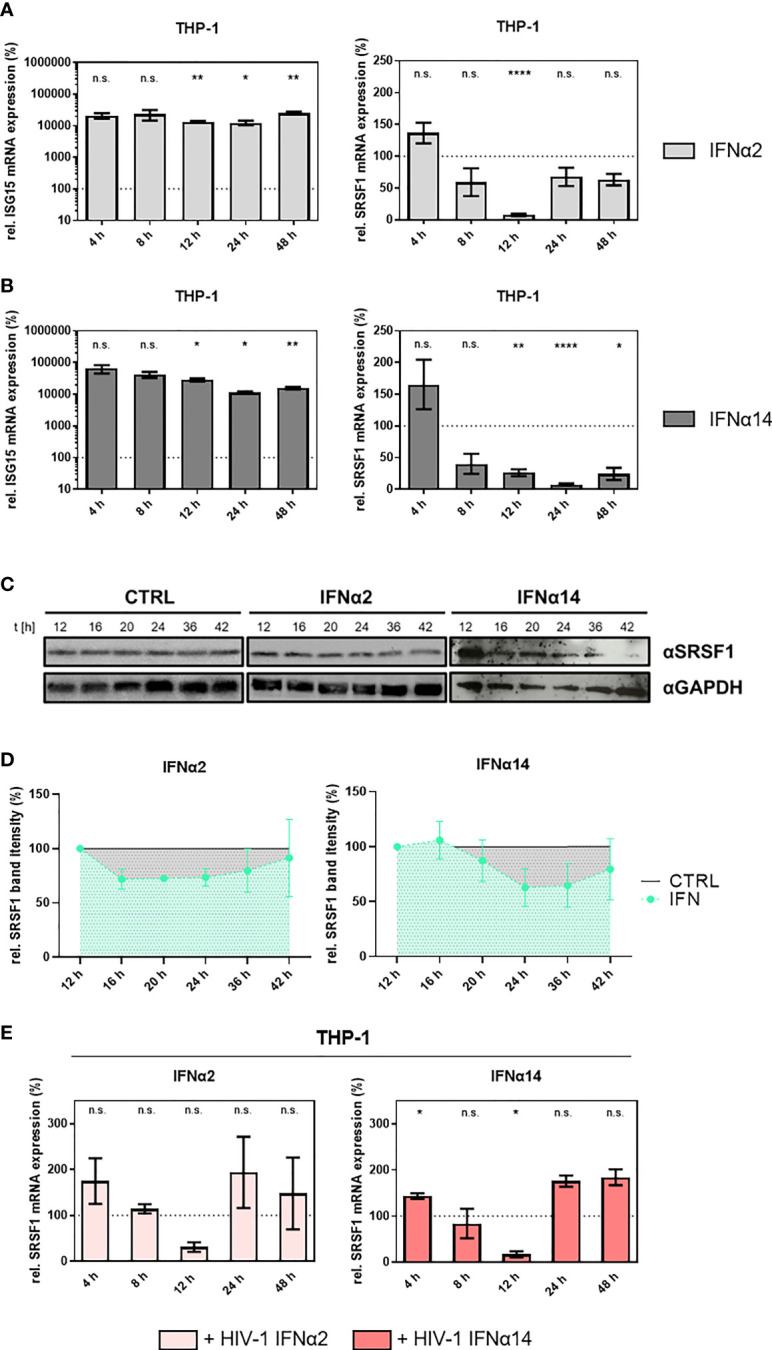
*SRSF1* mRNA and protein levels are differentially regulated upon stimulation of macrophages with IFNα2 or IFNα14. Differentiated THP-1 macrophages were treated with IFNα2 (light gray) or IFNα14 (dark gray) (10 ng/ml) over a period of 48 h before cells were harvested and RNA was isolated. Relative mRNA expression levels were measured *via* RT-qPCR analysis. **(A, B)** Relative mRNA expression levels of *ISG15* and *SRSF1* upon stimulation with **(A)** IFNα2 or **(B)** IFNα14 in THP-1 cells. Mixed-effects analyses followed by Dunnett’s *post-hoc* test were used to compare differences between groups at different time points (*p < 0.05, **p < 0.01, ****p < 0.0001 and ns, not significant). **(C)** Differentiated THP-1 macrophages were treated with IFNα2 or IFNα14 (10 ng/ml) for the indicated amount of time before cells were harvested and proteins were isolated. Proteins were separated by SDS-PAGE, blotted, and analyzed with an antibody specific to SRSF1. A representative Western blot is shown using GAPDH as a loading control. **(D)** Quantification of multiple Western blots (*n* = 4 for IFNa14, *n* = 3 for IFNa2). Total protein amount was stained using Trichloroethanol and used as loading control. Band intensity was measured using ImageJ software. Mixed-effects analyses followed by Dunnett’s *post-hoc* test. **(E)** Differentiated THP-1 macrophages were infected with the R5-tropic NL4-3 (AD8) (95) at an MOI of 1. Sixteen hours post-infection, cells were treated with the indicated IFN subtype (10 ng/ml) over a period of 48 h. Cells were harvested at the indicated time points, and RNA was isolated and subjected to RT-qPCR. Relative mRNA expression levels of SRSF1 in THP-1 cells after treatment with IFNα2 or IFNα14 as indicated. GAPDH was used as a house-keeping gene for normalization. Mixed-effects analyses followed by Dunnett’s *post-hoc* test were used to determine whether the difference between the group of samples reached the level of statistical significance (*p < 0.05, **p < 0.01, ***p < 0.001 and ****p < 0.0001). Mean ( ± SEM) of *n* = 4 biological replicates is shown.

To further assess the suggested role of *SRSF1* acting as IRepG, we examined *SRSF1* expression levels in HIV-1 target cells upon IFN stimulation in a time-course experiment. In differentiated THP-1 macrophages, we observed a strong 100- to 1,000-fold induction of *ISG15* mRNA expression levels remaining high up to 48 h post-stimulation with IFNα2 and IFNα14 (**Figures 4A, B**, left panel). Treatment with IFNα2 resulted in a 13-fold and highly significant downregulation of *SRSF1* after 12 h while expression levels were restored (less than twofold) 24–48 h post-treatment (**Figure 4A**, right panel). Treatment with IFNα14 also resulted in a significant 13-fold downregulation of *SRSF1* after 24 h, but for this subtype, a longer-lasting effect with a still 6-fold significant downregulation was observed after 48 h (**Figure 4B**, right panel). Overall, IFNα14 induced a stronger and more continuous repression than IFNα2. Interestingly, stimulation with both IFNα2 and IFNα14 induced an initial increase of 2.2- and 1.7-fold, respectively, in *SRSF1* mRNA expression 4 h post-treatment, albeit not statistically significant (**Figures 4A, B**, right panel).

To further analyze whether the IFN-induced reduction of SRSF1 could also be observed on protein level, we performed Western blot analysis of IFN-treated THP-1 cells. Both stimulation with IFNα2 and IFNα14 resulted in a time-dependent decrease in SRSF1 protein levels (**Figures 4C, D**). While treatment with IFNα2 only led to a weak repression, treatment with IFNα14 resulted in a more pronounced and longer-lasting downregulation of SRSF1 protein levels (**Figure 4C**), which was in accordance with the mRNA expression levels (**Figures 3A** and **4A, B**, right panel). When compared to the mRNA levels, SRSF1 protein levels decreased with a delay of 8-12 h, which might be explained by the half-life of persisting mRNA and protein levels. In agreement with the previous RNA data, the reduction in protein levels was observed earlier for IFNα2- than for IFNα14-treated cells. After the initial reduction, the protein levels were restored almost to initial levels at 42 h post-stimulation. Remarkably, IFNα14-treated cells tend to have a slow and more pronounced decrease in SRSF1 levels. However, this reduction was not significant due to the variability of the Western blotting procedure (**Figure 4D**). The protein amount continuously declined 16 h post-IFNα14 induction and was restored up to 60% of the initial level 42 h post-stimulation.

Next, we were interested whether a previous HIV-1 infection would affect the IFN-mediated time-dependent regulation of *SRSF1*. Therefore, we infected THP-1 macrophages with the R5-tropic HIV-1 laboratory strain NL4-3 AD8 16 h before stimulation with IFNα2 or IFNα14. This time point was specifically chosen to focus on HIV-1 post-integration steps, as the integration of the viral genome into the host cell genome is mostly completed (96). In infected THP-1 macrophages, treatment with IFNα2 and IFNα14 induced a comparable profile of *SRSF1* mRNA expression (**Figure 4E**). An initial upregulation, which was only significant for IFNα14, was observed after 4 h by 1.7- and 1.4-fold for IFNα2 and IFNα14, respectively. Treatment with IFNα2 resulted in a nonsignificant (*p* = 0.0522) 3.3-fold decrease in *SRSF1* mRNA levels after 12 h in HIV-1-infected cells (**Figure 4E**). Stimulation with IFNα14 resulted in a significant (*p* = 0.0139) 5.9-fold reduced SRSF1 expression after 12 h, which was restored after 24 and 48 h (**Figure 4E**).

While a 13-fold repression was observed in uninfected cells upon stimulation with IFNα2 and IFNα14 after 12 and 24 h, respectively, stimulation of HIV-1-infected cells only resulted in a 3.3-fold (IFNα2) and 5.9-fold (IFNα14) decrease in *SRSF1* mRNA levels 12 h post-treatment. Thus, the magnitude of repression was stronger in uninfected cells when compared to HIV-1-infected cells. While the *SRSF1* expression profile upon stimulation with IFNα2 and IFNα14 was different in uninfected cells, no large discrepancies were observed upon treatment of HIV-1-infected cells with both subtypes. Contrary to uninfected cells, *SRSF1* mRNA expression was restored already 24 h post-stimulation of HIV-1-infected cells with IFNa14.

Since viral proteins were shown to modulate the expression of cellular genes (97–101), we next overexpressed a panel of HIV-1 accessory proteins in HEK293T cells. Consistent with previous literature (97, 98), we confirmed that the viral protein Vpr was able to significantly increase ISG15 expression (**Supplementary Figure 3**). Further analysis with a full-length viral genome and a Vpr-deficient derivative confirmed the significant involvement of Vpr in an infectious context. Since an inverse correlation between ISG induction and *SRSF1* repression has been observed, Vpr could potentially be directly involved in the downregulation of SRSF1 observed upon HIV-1 infection.

**Figure 5:**
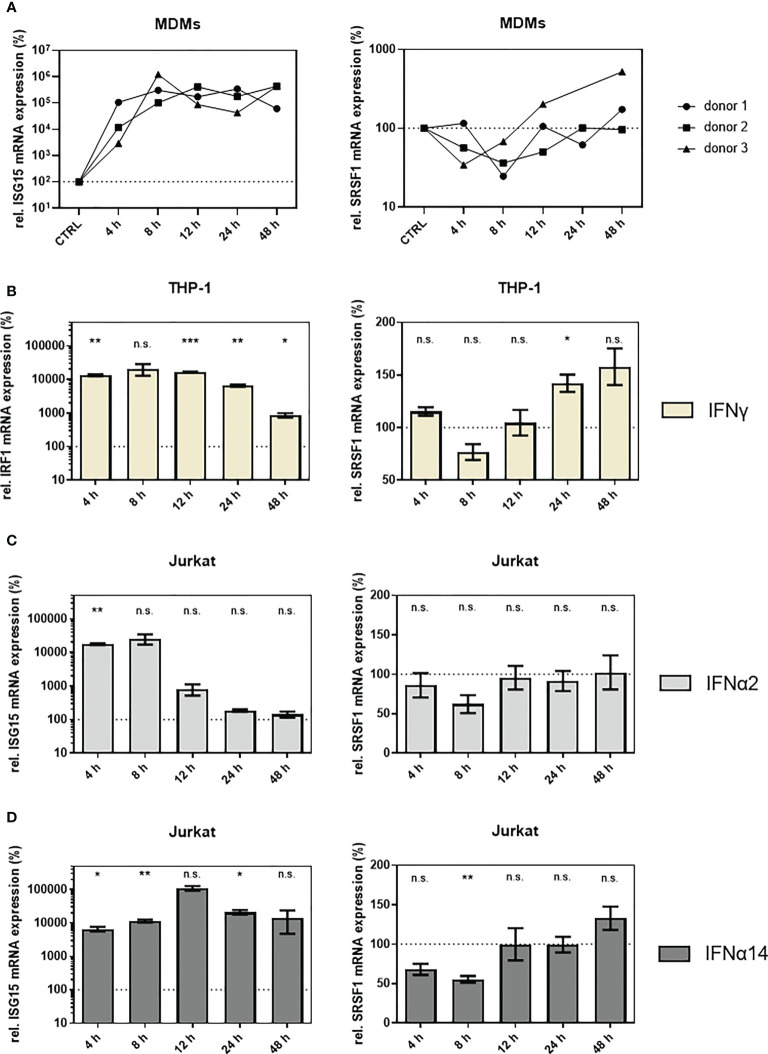
*SRSF1* mRNA levels are differentially regulated upon treatment of HIV-1 target cells with IFNs. **(A)** Monocyte-derived macrophages (MDMs) were treated for 48 h with 10 ng/ml of IFNα14. After harvesting the cells, RNA was extracted and subjected to RT-qPCR. Relative mRNA expression levels of *ISG15* and *SRSF1* are shown. Expression levels were normalized to GAPDH. Time point of 24 h only includes two biological replicates. **(B)** Differentiated THP-1 cells were stimulated with IFNγ (10 ng/ml) for 48 h before cells were harvested and RNA was isolated. Relative mRNA expression levels of *IRF1* and *SRSF1* were measured *via* RT-qPCR. GAPDH was used as a house-keeping gene for normalization. Mixed-effects analysis followed by Dunnett’s *post-hoc* test was conducted to determine whether the difference between the group of samples reached the level of statistical significance (*p < 0.05, **p < 0.01, ***p < 0.001, and ns, not significant). Mean ( ± SEM) of *n* = 4 biological replicates is shown. **(C, D)** Relative mRNA expression levels of *ISG15* and *SRSF1* upon stimulation with **(C)** IFNα2 or **(D)** IFNα14 in Jurkat cells. ACTB was used as a house-keeping gene for normalization. Mixed-effects analysis with Dunnett’s *post-hoc* test was performed to determine whether the difference between the group of samples reached the level of statistical significance (*p < 0.05, **p < 0.01, ***p < 0.001, and ns, not significant). Mean ( ± SEM) of *n* = 4 biological replicates is shown.

To assess, whether these findings could be transferred to primary human cells, we analyzed gene expression of *SRSF1* after stimulation of primary human monocyte-derived macrophages (MDMs) with IFNα14. A strong 50- to 500-fold induction was observed for *ISG15* mRNA expression levels in all three tested donors (**Figure 5A**, left panel). Concomitantly, a time-dependent repression of *SRSF1* mRNA was detected with a 1.5-fold (donor 3), 2.8-fold (donor 2), and 4.1-fold (donor 1) downregulation after 8 h (**Figure 5A**, right panel), thus supporting a potential role of *SRSF1* as IRepG in primary human macrophages. Of note, the IFNα14-induced downregulation of *SRSF1* mRNA levels was less pronounced in MDMs than in THP-1 macrophages. While IFNα14 induced a long-lasting and strong repression of *SRSF1* mRNA expression in THP-1 macrophages, *SRSF1* mRNA levels in MDMs were comparable to the untreated control already after 12–24 h.

To analyze whether the downregulation of *SRSF1* was IFN-I specific, we included IFNγ as the sole member of the type II IFN family (IFN-II) (102, 103). Since IFNγ binds to the IFNγ receptor (IFNGR) and activates a distinct signaling pathway than IFN-I (8, 102, 104), IFN-regulatory factor 1 (IRF1) was chosen as a control of IFN-II-specific activation of the IFNγ activation site (GAS) regulated promotor (105, 106). Stimulation of THP-1 macrophages with IFNγ led to a strong 50- to 100-fold induction of *IRF1* mRNA 4–24 h after stimulation. After 48 h, only a remaining ninefold induction in *IRF1* mRNA expression was observed (**Figure 5B**, left panel). IFNγ treatment only induced a weak and nonsignificant 1.3-fold downregulation in *SRSF1* mRNA expression levels after 8 h. Remarkably, 12–48 h post-stimulation, a time-dependent increase in *SRSF1* mRNA was observed with significantly elevated levels after 24 h (1.4-fold) (**Figure 5B**, right panel). Hence, the IFN-mediated regulation of *SRSF1* mRNA expression seems to also involve IFN-II signaling.

In Jurkat T cells, treatment with IFNα2 led to a significant 100-fold induction in *ISG15* mRNA expression levels after 4 h, while 12-h post-stimulation *ISG15* levels drastically dropped to the levels comparable to PBS-treated control cells (**Figure 5C**, left panel). Treatment with IFNα14 resulted in a continuous (24 h) 100- to 1,000-fold significant induction of *ISG15* mRNA expression levels (**Figure 5D**, left panel). Treatment with IFNα14 resulted in twofold (*p* = 0.0057) repression after 8 h (**Figure 5D**, right panel). *SRSF1* mRNA expression levels were restored 12 h post-stimulation (**Figure 5D**, right panel).

Thus, a time-dependent downregulation in *SRSF1* mRNA levels was observed in HIV-1 target cells upon treatment with IFNα14, suggesting a potential role of *SRSF1* as an IRepG. Since the repression of *SRSF1* mRNA expression was much more pronounced in THP-1 macrophages than in Jurkat T cells, a cell-type-specific effect was suggested.

### Deregulation of SRSF1 expression occurs on RNA level

**Figure 6:**
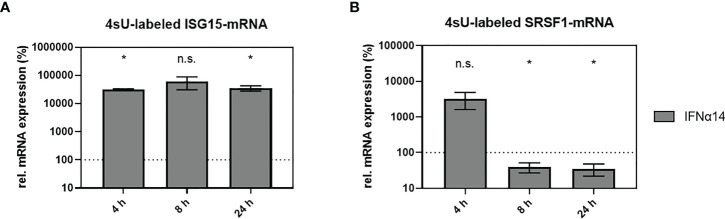
IFNα14-mediated changes in newly transcribed *SRSF1* mRNA. **(A, B)** THP-1 macrophages were stimulated with IFNα14 (10 ng/ml). 4-Thiouridine (4sU) was added 30 min before harvesting the cells at the indicated time points in order to label newly synthesized RNA. After separation and isolation of the freshly synthesized RNA, RT-qPCR was performed to measure relative mRNA expression levels of **(A)** *ISG15* and **(B)** *SRSF1*. GAPDH was used as a house-keeping gene for normalization. Statistical significance compared to untreated control was determined using unpaired two-sided Welch’s t-test. Asterisks indicated *p*-values as *p < 0.05 and ns, not significant. Mean ( ± SEM) of *n* = 3 biological replicates is shown (except for 4 h, where *n* = 2).

*SRSF1* was identified as an IFN-regulated gene in HIV-1 target cells; however, the mechanistic mode of action was still unknown. To assess whether the IFN-mediated deregulation of *SRSF1* mRNA expression occurred on the transcriptional level, we used the method of 4-thiouridine (4sU)-tagging (30, 107–110). This method allows the metabolic labeling of freshly transcribed RNA using 4sU, enabling the subsequent purification and separation of newly synthesized RNA from untagged pre-existing RNA. THP-1 macrophages were treated with IFNα14 for 4, 8, or 24 h and 4sU was added 30 min prior to cell harvest. Following the biotinylation of the incorporated 4sU, the freshly transcribed RNA was separated from the unlabeled RNA using Streptavidin-coated magnetic beads. Changes in transcription rates were measured *via* RT-qPCR. Comparison to the untreated control stimulation with IFNα14 resulted in a significant increase in *ISG15* mRNA expression after 4 and 24 h (319- and 352-fold), respectively (**Figure 6A**). Remarkably, a 33-fold, but nonsignificant, increase in *SRSF1* mRNA expression was observed 4 h post-treatment (**Figure 6B**). A significant 2.5-fold reduction in *SRSF1* mRNA expression was observed after 8 h, which was still significantly reduced by 2.9-fold after 24 h (**Figure 6B**).

These data indicate that the IFN-mediated regulation of *SRSF1* likely occurs on the transcriptional level. The expression profile of *SRSF1* analyzing 4sU-labeled and freshly transcribed RNA was in accordance with the expression profile of *SRSF1* analyzing total RNA (**Figure 4B**, right panel). Both data sets revealed a multi-phasic pattern with an early increase in mRNA expression followed by a strong downregulation.

### Knockdown of SRSF1 levels affect HIV-1 post-integration steps

SRSF1 has been described as a crucial regulator in HIV-1 post-integration steps. In addition to SRSF1 regulating alternative splice site usage *via* targeting several binding sites on the viral pre-mRNA (78, 81), SRSF1 was also described to compete with binding of HIV-1 Tat to a sequence within the TAR region, thereby impeding HIV-1 LTR activity (83).

**Figure 7:**
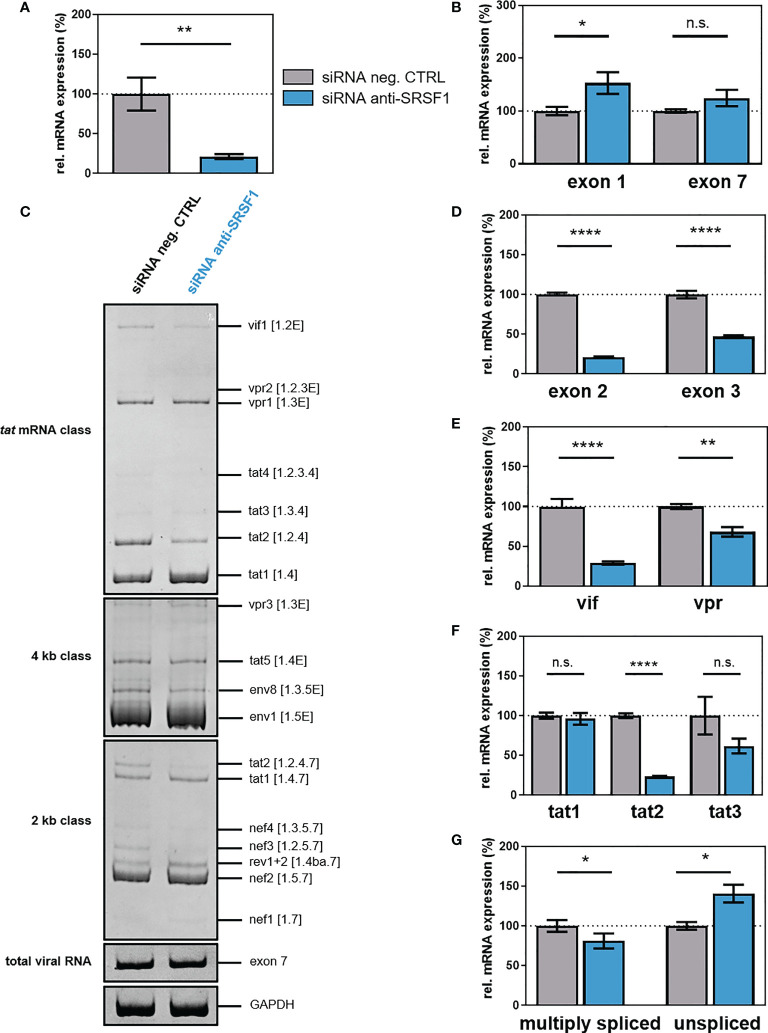
siRNA-mediated knockdown of SRSF1 affects HIV-1 LTR transcription and splice site usage. HEK293T cells were transfected with the proviral clone pNL4-3 PI952 and anti-SRSF1 siRNA or an siRNA negative control. Seventy-two hours post-transfection, cells were harvested and RNA and viral supernatant was isolated. Isolated RNA was subjected to RT-qPCR. **(A, B)** Relative mRNA expression levels of **(A)** *SRSF1* and **(B)** exon 1- and exon 7-containing mRNAs (total viral mRNA) normalized to GAPDH expression. **(C)** Analysis of viral splicing pattern upon SRSF1 knockdown. Isolated RNA was subjected to RT-PCR analysis using the indicated primer pairs for the 2-kb, 4-kb, and *tat* mRNA class (**Supplementary Table 1**, **Supplementary Table 2**, and **Supplementary Figure 4**). HIV-1 transcript isoforms are depicted on the right according to Purcell and Martin (36). To compare total RNA amounts, separate RT-PCRs amplifying HIV-1 exon 7-containing transcripts as well as cellular GAPDH were performed. PCR amplicons were separated on a 12% non-denaturing polyacrylamide gel. **(D–G)** Relative expression levels of **(D)** exon 2- and exon 3-containing, **(E)** *vif* and *vpr*, **(F)** *tat1*, *tat2*, and *tat3*, and **(G)** multiply spliced and unspliced mRNAs. HIV-1 mRNAs were analyzed using the indicated primers (**Supplementary Table 1**, **Supplementary Table 2**, and **Supplementary Figure 4**). The mRNA expression of NL4-3 PI952 was set to 100%, and the relative splice site usage was normalized to total viral mRNA levels (exon 7-containing mRNAs). Unpaired two-tailed *t*-tests were calculated to determine whether the difference between the group of samples reached the level of statistical significance (*p < 0.05, **p < 0.01, ****p < 0.0001 and ns, not significant). Mean ( ± SEM) of *n* = 4 biological replicates is depicted for **(A)**, **(B)**, and **(D–G)**. For **(C)**, a representative gel is shown.

To evaluate the impact of IFN-mediated repression of *SRSF1* on HIV-1 post-integration steps, we transiently silenced endogenous SRSF1 expression using a siRNA-mediated knockdown approach. HEK293T cells were transiently co-transfected with a plasmid coding for the HIV-1 laboratory strain NL4-3 PI952 (pNL4-3 PI952) (111) and an SRSF1-specific siRNA or a siRNA negative control. Seventy-two hours post-transfection, cells and the virus-containing supernatant were harvested. Knockdown efficiency was verified *via* One-Step RT-qPCR, revealing a significant knockdown of *SRSF1* gene expression of 85% when compared to the negative control siRNA (**Figure 7A**). Intracellular total viral mRNA levels were determined by means of exon 1- or exon 7-containing mRNAs, which are present in all viral mRNA isoforms (36). Upon knockdown of SRSF1, exon 1-containing mRNA transcripts were significantly increased by 1.5-fold (**Figure 7B**). Thus, depleted levels of SRSF1 could potentially result in less competition in TAR binding, thereby increasing LTR promoter activity.

Next, we investigated whether knockdown of SRSF1 might affect HIV-1 alternative splicing and performed semi-quantitative RT-PCR focusing on viral intron-less (2 kb) and intron-containing (4 kb) mRNA classes as well as *tat*-specific mRNA isoforms. No changes in the viral splicing pattern were observed in the 4-kb mRNA class upon depleted levels of SRSF1 (**Figure 7C**). In the 2-kb mRNA class, *tat2* and *nef3* mRNA expression was reduced, while in the *tat*-specific mRNA class, *vif1* and *tat2* levels were decreased (**Figure 7C**). The formation of *vif1*, *tat2*, and *nef3* mRNA, which all contain non-coding leader exon 2, requires splicing from splice donor (SD) 1 to splice acceptor (SA) 1 (36). Thus, an effect of depleted levels of *SRSF1* on the frequency of SA1 splice site recognition and usage was suggested. Alterations in the expression of specific HIV-1 mRNA transcripts were also quantitatively confirmed by RT-qPCR using transcript-specific primer pairs (**Figures 7D–G**, **Supplementary Table 1**, **Supplementary Table 2**, **Supplementary Figure 4**). The frequency of transcripts containing the non-coding leader exon 2 and 3 was significantly reduced by 5- and 2-fold, respectively (**Figure 7D**). Knockdown of SRSF1 resulted in a 3.6-fold downregulation of *vif* mRNA expression (**Figure 7E**). Since it has previously been shown that reduced Vif protein levels result in viral replication failure (112–114), the substantial loss in *vif* mRNA could potentially severely affect viral replication. The formation of *vif* mRNA requires splicing from SA1 to SD1 (36). Thus, the observed repression in exon 2 inclusion and *vif* mRNA levels suggested a reduced splicing frequency at SA1. Since the SRE ESE M1/M2 was shown to regulate splice site usage at SA1 and is a known target of SRSF1 (79), it was suggested that lower levels of SRSF1 resulted in a lower recognition of SA1 and thus induced a decrease in *vif* and exon 2-containing mRNA transcripts. Furthermore, *vpr* mRNA expression was decreased by 1.4-fold (**Figure 7E**). Splicing from SD1 to SA2 is required for the generation of *vpr* mRNA (36). The observed reduction in exon 3 inclusion and *vpr* mRNA expression suggested a reduced splice site usage at SA2. However, no SRSF1-bound SRE involved in the regulation of SA2 splice site usage has yet been identified. While mRNA levels of *tat1* were not altered upon SRSF1 knockdown, both *tat2* and *tat3* mRNAs were repressed by four- and twofold, respectively; however, only the reduction of tat2 was significant (**Figure 7F**). Splicing from SD4 to SA7 is required for the formation of *tat1*, *tat2*, and *tat3* mRNA. Furthermore, splicing from SD1 to SA3 (*tat1* mRNA), from SD1 to SA1 and SD2 to SA3 (*tat2* mRNA), and from SD1 to SA2 and SD3 to SA3 (*tat3* mRNA) is necessary for the generation of the respective mRNAs (36). Since the levels of multiply spliced mRNAs (spliced from SD4 to SA7) were significantly decreased by 1.3-fold, while the levels of unspliced viral mRNAs (containing intron 1) were significant increased by 1.4-fold, knockdown of SRSF1 was suggested to shift the ratio towards unspliced mRNAs (**Figure 7G**). The suggested decrease in splicing events at SA1 and SA2 were in accordance with the increased amount of unspliced mRNAs. The decrease in multiply spliced mRNAs indicates reduced SD4–SA7 splicing, suggesting a possible involvement of the SRSF1-bound SRE ESE3, which regulates splicing at SA7 (80).

**Figure 8:**
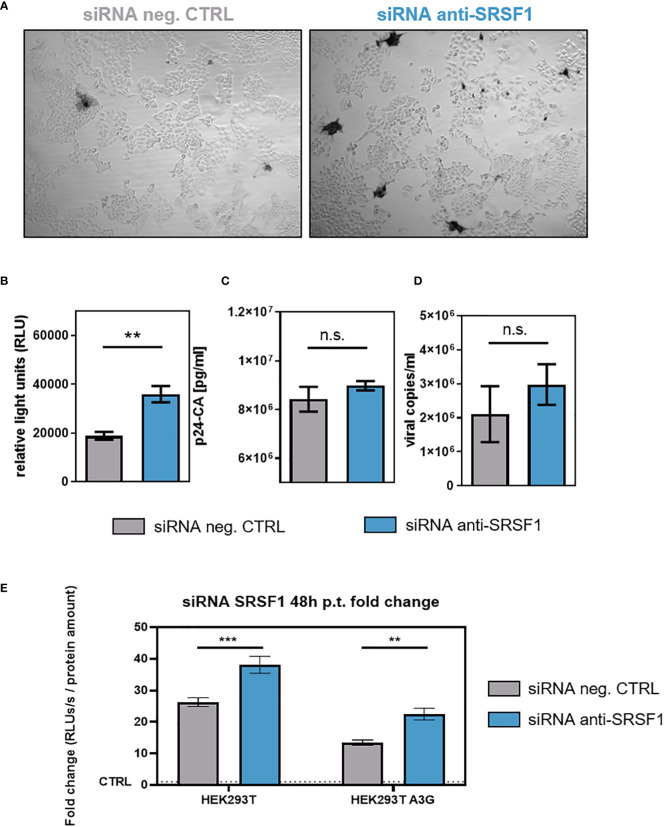
Impact of siRNA-based knockdown of SRSF1 on HIV-1 infectivity and virus production. RPE-ISRE luc cells were transfected with a plasmid coding for the proviral clone NL4-3 (AD8) (pNL4-3 AD8) and the indicated siRNA. Seventy-two hours post-transfection, cellular supernatant was harvested. **(A, B)** Viral infectivity was determined *via* TZM-bl assay. **(A)** Infected TZM-bl cells were stained with X-Gal. **(B)** Luciferase activity was determined measuring relative light units (RLUs). **(C)** p24-CA ELISA was performed to determine p24-CA levels in the cellular supernatant. **(D)** Cellular supernatant was used to determine viral copy number per milliliter. RT-qPCR was performed analyzing absolute expression levels of exon 7-containing transcripts (total viral mRNA). Statistical significance was determined using unpaired two-tailed *t*-tests (**p < 0.01). Mean ( ± SEM) of *n* = 4 biological replicates is shown for **(B)**, **(C)**, and **(D)**. **(E)** HEK293T- and HEK293T APOBEC3G-expressing cells were seeded in poly-L-lysine (Sigma-Aldrich) pre-coated wells. Cells were transiently transfected with the proviral clone pNL4-3 and 12.8 nM of the indicated siRNA. Supernatants were harvested 48 h post-transfection and applied to TZM-bl cells. Forty-eight hours post-infection, cells were lysed for luciferase assay. The RLUs/s were normalized to whole protein amounts as determined using Bradford assay. The fold change is normalized to the signal of uninfected TZM-bl cells. Mean ( ± SEM) of *n* = 4 biological replicates and *n* = 2 technical replicates is shown. The significance was analyzed using two-way ANOVA (**p < 0.01, ***p < 0.001, and ns, not significant).

To investigate whether SRSF1 knockdown and the observed effects on HIV-1 LTR transcription and alternative splice site usage would affect viral infectivity, RPE-ISRE luc reporter cells were transiently co-transfected with a plasmid expressing the HIV-1 laboratory strain NL4-3 (pNL4-3) (115) and a SRSF1-specific siRNA or a siRNA negative control. Forty-eight hours post-transfection supernatants of transfected cells were collected. TZM-bl cells were then infected with virus-containing cellular supernatant. TZM-bl cells harbor reporter genes for firefly luciferase and β-galactosidase under the control of the HIV-1 LTR promoter (116). Since the LTR-dependent luciferase activity and β-galactosidase of the reporter cells was significantly increased by up to 1.7-fold, low SRSF1 levels were facilitating viral infectivity (**Figures 8A, B**). Interestingly, the observed changes in HIV-1 splice site usage, including a 3.6-fold reduction in *vif* mRNA expression (**Figure 7E**), did not impede viral infectivity.

Only a marginal and nonsignificant 1.1-fold increase in p24-CA levels was detected upon SRSF1 knockdown (**Figure 8C**). RT-qPCR analysis with viral RNA extracted from the cellular supernatant was performed, revealing a 1.4-fold increase in viral copies, albeit not significant (**Figure 8D**).

To demonstrate the effect of Vif reduction and increased LTR activity in the presence of the restriction factor APOBEC3G, HEK293T- and APOBEC3G-expressing derivate cells were co-transfected with HIV-1 encoding plasmid DNA and siRNA against SRSF1 (**Figure 8E**). The virus-containing supernatants were used to infect TZM-bl reporter cells. Compared to parental cells, the presence of APOBEC3G significantly reduced the infectivity of the virus particles. Interestingly, however, SRSF1 knockdown still led to a significant increase in infectivity (**Figure 8E**), which was in agreement with previous observations (**Figure 8B**).

In conclusion, although siRNA-mediated knockdown of SRSF1 predominantly lowered *vif* mRNA expression, low SRSF1 levels resulted in facilitated viral infectivity, most likely due to increased LTR promoter activity. Thus, suppression of SRSF1 levels, as observed in the early immune response to an HIV-1 infection, could potentially facilitate HIV-1 infectivity and the establishment of a systemic infection.

### Overexpression of SRSF1 levels negatively affects HIV-1 replication

**Figure 9:**
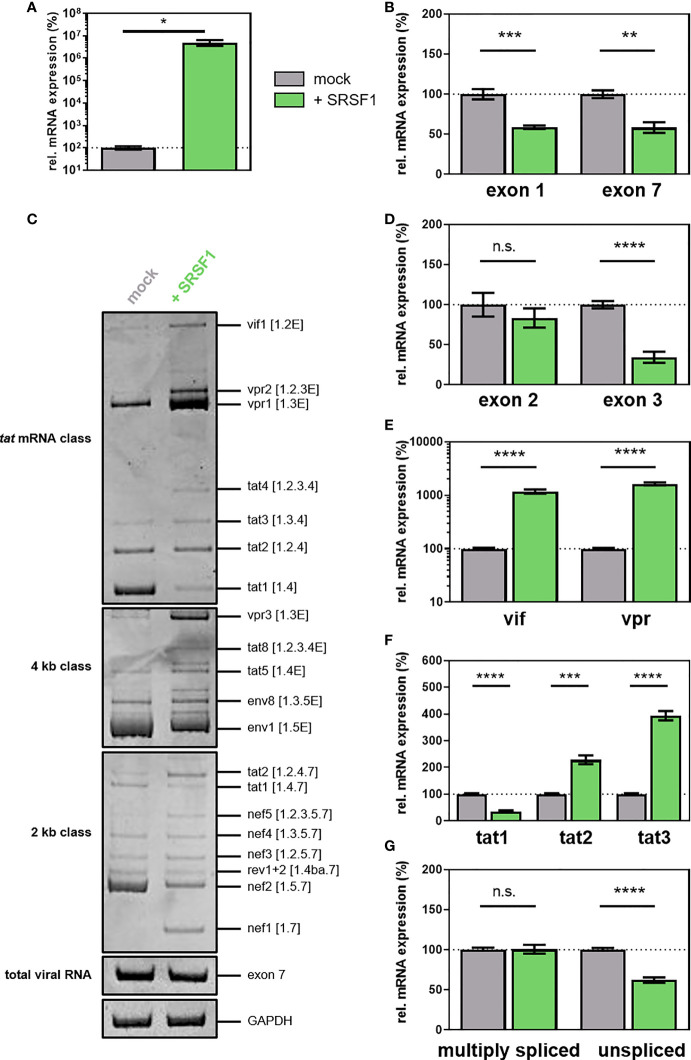
**(A–G)** Overexpression of SRSF1 affects HIV-1 LTR transcription and alternative splice site usage. HEK293T cells were transiently transfected with a plasmid coding for the proviral clone NL4-3 PI952 (pNL4-3 PI952) and a plasmid expressing FLAG-tagged SRSF1 (pcDNA-FLAG-SF2) or an empty vector [pcDNA3.1(+)] as mock control. Cellular RNA and cell culture supernatant were harvested 72 h post-transfection and subjected to further analysis. **(A, B)** RT-qPCR was performed to determine relative mRNA expression levels of **(A)** *SRSF1* and **(B)** exon 1- and exon 7-containing mRNAs (total viral mRNA). GAPDH was used for normalization. **(C)** RT-PCR was performed using the indicated primer pairs covering the viral mRNA isoforms of the 2-kb, 4-kb, and *tat* mRNA class (**Supplementary Tables 1**, **2** and **Supplementary Figure 4**). HIV-1 transcript isoforms are indicated according to Purcell and Martin (36). HIV-1 exon 7-containing transcripts as well as cellular GAPDH were included as loading controls. PCR amplicons were separated on a 12% non-denaturing polyacrylamide gel. **(D, G)** Total RNA was subjected to RT-qPCR to measure relative mRNA expression levels of **(D)** exon 2- and exon 3-containing, **(E)** *vif* and *vpr*, **(F)** *tat1*, *tat2*, and *tat3*, and **(G)** multiply spliced and unspliced mRNAs using the indicated primers. Relative viral splice site usage was normalized to exon 7-containing mRNAs (total viral RNA). Unpaired two-tailed *t*-tests were used to calculate statistical significance (*p < 0.05, **p < 0.01, ***p < 0.001, and ****p < 0.0001). Mean ( ± SEM) of *n* = 4 biological replicates is depicted for **(A, B)** and **(D–G)**. For **(C)**, a representative gel is shown. ns is not significant.

IFNα14 stimulation of THP-1 cells and primary macrophages resulted in SRSF1 repression (**Figures 4**–**6**) and higher infectivity (**Figures 7** and **8**). To assess whether elevated levels of SRSF1 would negatively affect HIV-1 post-integration steps, we transiently transfected HEK293T cells with a plasmid expressing an HIV-1 laboratory strain (pNL4-3 PI952) (111) and an expression plasmid coding for FLAG-tagged SRSF1 (pcDNA-FLAG-SF2) (117) or an empty vector [pcDNA3.1(+)] as mock control. After 72 h, cellular RNA and virus-containing supernatant were harvested. One-Step RT-qPCR analysis revealed enhanced levels of *SRSF1* by multiple orders of magnitude (**Figure 9A**). Furthermore, protein expression and nuclear localization were confirmed using immune fluorescence microscopy (**Supplementary Figure 5**). As determined by RT-qPCR using primer pairs amplifying exon 1- or exon 7-containing mRNAs (**Supplementary Figure 4**), overexpression of SRSF1 induced a significant 1.7-fold reduction in total viral mRNA levels (determined by both exon 1- and exon 7-containing mRNAs) (**Figure 9B**). Increased levels of SRSF1 were thus suggested to inhibit LTR transcription, which was in accordance with previous data (78, 82).

Examining the viral splicing patterns *via* semi-quantitative RT-PCR, significant changes in all HIV-1 mRNA classes were observed upon overexpression of SRSF1 (**Figure 9E**). Increased levels of *vif1*, *vpr1-2*, and *tat2-4* (tat mRNA class); *tat2*, *nef1*, and *nef5* (2-kb class); and *vpr3*, *tat5*, and *tat8* (4-kb class) were detected, while the transcript levels of *tat1* (tat mRNA class), *tat1* and *nef2* (2-kb class), and *env1* (4-kb class) were reduced (**Figure 9C**). Alterations in the expression of specific HIV-1 mRNA transcripts were also quantitatively confirmed by RT-qPCR using transcript-specific primer pairs (**Figures 9D–G**; **Supplementary Tables 1**, **2**, **Supplementary Figure 4**). While the inclusion of non-coding leader exon 2 was not altered upon elevated levels of SRSF1, the inclusion of non-coding leader exon 3 was significantly decreased by threefold (**Figure 9D**). The mRNA expression levels of *vif* (spliced from SD1 to SA1) and *vpr* (spliced from SD1 to SA2), which crucially depend on the recognition of SA1 and SA2, were strongly increased by 12- and 16-fold, respectively (**Figure 9E**). These findings suggested that the splicing frequency at SA1 and SA2 was substantially increased, whereas splicing at SD2 and SD3 was inhibited. Since cross-exon interactions play a crucial role in exon 2 and exon 3 definition, it is likely that elevated levels of SRSF1 affect cross-exon interactions between SA1 and SD2, and SA3 and SD3. Furthermore, overexpression of SRSF1 induced a threefold decrease in *tat1* mRNA levels, while the levels of *tat2* and *tat3* mRNA, which are also dependent on SA1 and SA2 recognition, increased by 2.3- and 4-fold, respectively (**Figure 9F**). The increased splice site usage at SA1 and SA2 was in accordance with the observed 1.6-fold decrease in unspliced mRNA transcripts (**Figure 9G**). An effect of elevated SRSF1 levels on the splicing frequency at SA7 was unlikely since the levels of multiply spliced mRNAs (spliced from SD4 to SA7) were not significantly altered (**Figure 9G**).

**Figure 10:**
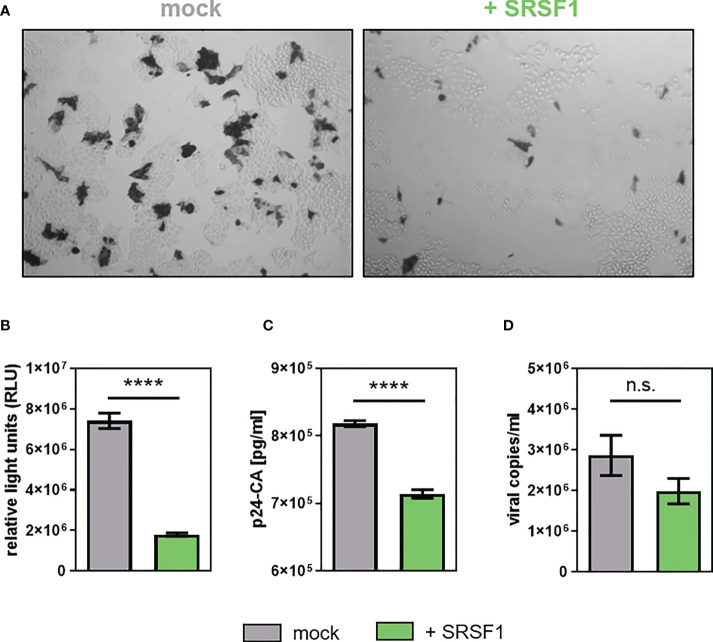
Overexpression of SRSF1 affects HIV-1 infectivity and viral particle production. HEK293T cells were co-transfected with a plasmid coding for the proviral clone NL4-3 PI952 (pNL4-3 PI952) (371) and a plasmid coding for FLAG-tagged SRSF1 (pcDNA-FLAG-SF2) or an empty vector [pcDNA3.1(+)] as mock control. **(A, B)** Seventy-two hours post-transfection, the cell culture supernatant was used to determine viral infectious titers using TZM-bl reporter cells. **(A)** X-Gal staining of TZM-bl cells incubated with the cellular supernatant. **(B)** Measurement of luciferase activity. **(C)** Viral RNA extracted from the supernatant was subjected to RT-qPCR to quantify absolute expression levels of exon 7-containing transcripts (total viral mRNA). **(D)** p24-CA ELISA was performed to determine viral particle production. Statistical significance was calculated using unpaired two-tailed *t*-tests (****p < 0.0001 and ns, not significant). Mean ( ± SEM) of *n* = 4 biological replicates is depicted for **(B–D)**.

To analyze whether overexpression of SRSF1 and the concomitant changes in HIV-1 LTR transcription and alternative splice site usage would affect viral infectivity, TZM-bl reporter cells were infected with the virus-containing cellular supernatant. High levels of SRSF1 resulted in a strong decrease in infectious viral titers by X-Gal staining of infected TZM-bl cells (**Figure 10A**). As confirmed with a quantitative luciferase assay, a 4.1-fold significantly lower infectivity was observed (**Figure 10B**).

The levels of p24-CA were significantly lower (1.2-fold) upon SRSF1 overexpression (**Figure 10C**). The number of viral copies in the supernatant was determined *via* RT-qPCR analysis, revealing 1.4-fold lower (but not significant) levels when compared to the mock control (**Figure 10D**).

In conclusion, plasmid-driven overexpression of SRSF1 substantially altered the balanced ratios of HIV-1 mRNA transcripts and negatively affected Tat-LTR transcription. Contrary to reduced SRSF1 levels, overexpression significantly impaired HIV-1 particle production and infectivity.

## Discussion

Type I IFNs (IFN-I) play a key role in the early immune defense against viral infections (1, 2). Their mode of action includes immunomodulatory functions and the upregulation of hundreds of IFN-stimulated genes (ISGs), thereby establishing an antiviral state in infected host and bystander cells. Furthermore, they induce the downregulation of host factors essential for viral replication, which are termed IRepGs (29, 30). In this study, we were able to identify the cellular splicing factor *SRSF1* as an IFN-I-regulated gene, crucially affecting HIV-1 post-integration steps at the level of LTR transcription, alternative splicing, and virus production.

Based on the increased ISG expression in PBMCs (**Figure 2A**) and LPMCs (87) of acutely and chronically HIV-1-infected individuals, we confirmed both of our patient cohorts as being representative. Interestingly, we found the *ISG15* mRNA expression to be lower in ART-treated patients when compared to HIV-1-infected treatment-naïve individuals in PBMCs (**Figure 2A**). Indeed, it has been shown previously that in LPMCs of patients under ART treatment, ISG levels were comparable to healthy donors (87). It was shown that more than 4,000 genes are differentially expressed (118) and the IFN-induced JAK-STAT signaling pathway and several ISGs, such as ISG15, Mx2, or IFITM1, were downregulated following ART treatment. However, whether the low ISG level in our ART-treated cohort might result from a decrease in inflammation or from the administered drug needs further investigation.

During our initial screen, we investigated differences in the expression levels of *SRSF* transcripts in LPMCs of healthy donors and HIV-1-infected individuals, either treatment-naïve or under ART treatment. Specific *SRSF* transcript levels, particularly *SRSF1*, were significantly lower upon HIV-1 infection (**Figure 1A**). These findings implied a direct or indirect effect of the HIV-1 infection on the gene expression of specific *SRSF* transcripts, potentially as a consequence of the IFN signaling. Supporting this hypothesis, a significant correlation was observed between the induction of *ISG15* mRNA expression and the repression of *SRSF1* mRNA levels in PBMCs of acutely HIV-1-infected patients (**Figure 2F**). Thus, the strong upregulation of IFN-I and subsequent induction of ISGs during the early inflammation in response to an HIV-1 infection (23) could potentially directly or indirectly result in the repression of *SRSF1*, suggesting a potential role of *SRSF1* as IRepG.

Interestingly, no significant differences were observed in the expression levels of most *SRSFs* when comparing LPMCs of ART-treated patients and healthy donors (**Figure 1B**). The expression levels of *SRSF1* mRNA, which were reduced by 2-fold upon HIV-1 infection (**Figure 1A**), were even elevated by 1.2-fold upon ART treatment when compared to uninfected donors. Remarkably, while *SRSF1* transcript levels were restored upon ART treatment in gut LPMCs, this effect was not observed in PBMCs, indicating a tissue-dependent specificity (**Figure 2C**). This discrepancy could possibly be explained through the phenotypical and functional differences between the two immune cell populations. PBMCs and LPMCs reside within a strongly differing micro-environment, include a different composition of immune cells, and express distinct cytokines and receptors (119–127).

A significant time-dependent repression in *SRSF1* mRNA levels was observed upon stimulation with IFN-I in THP-1 macrophages, Jurkat T cells, and MDMs (**Figures 4**, **5**). Since the effect on *SRSF1* mRNA expression varied greatly between cell types, and between transformed cell lines and primary cells, the IFN-I-mediated repression seemed to underlie cell-type-specific characteristics, such as the expression pattern of co-regulatory factors. The physiological relevance of primary human cells is certainly higher than transformed cell lines, since they provide a closer resemblance to physiological conditions (128, 129). Since treatment with IFNγ only led to a negligible effect on *SRSF1* mRNA expression in THP-1 macrophages when compared to IFNα2 or IFNα14, a preferentially IFN-I-specific effect was implied (**Figures 4**, **5**). However, while binding two distinct receptors and signaling *via* different pathways (2, 8), cross-talk between IFN-I and IFN-II has been observed (130–132).

The observed lower levels of *SRSF1* mRNA transcripts upon HIV-1 infection in LPMCs and PBMCs, as well as the time-dependent downregulation in *SRSF1* mRNA in various cell types upon stimulation with IFN-I, suggested *SRSF1* to represent an IRepG. However, prior to the strong downregulation upon stimulation with IFNα2 and IFNα14 in THP-1 macrophages, an increase in *SRSF1* mRNA expression was detected (**Figures 4A, B**, right panel). The method of 4sU tagging, which allows the analysis of RNA synthesis and degradation, as well as transcription factor functions (30, 108–110), confirmed a strong increase in *SRSF1* mRNA expression prior to the downregulation and revealed the changes in gene expression to most likely occur on the transcriptional level (**Figure 6**). Other regulatory mechanisms, however, might potentially be involved in the biphasic expression pattern. *SRSF1* was shown to be a direct transcriptional target of the oncogenic transcription factor Myc (133, 134). Since *Myc* expression was shown to be reduced by elevated levels of IFN-I (135–137), the observed reduction in *SRSF1* mRNA expression could potentially result from depleted Myc levels. Furthermore, SRSF1 was shown to maintain homeostasis through an autoregulatory feedback loop (138, 139). Since deregulation of SRSF1 was shown to be involved in tumorigenesis (133) (140, 141), tight regulation of *SRSF1* expression is important for normal cell physiology. The most abundant *SRSF1* transcript isoform is the productively translated and intron-containing isoform 1 (139). Upon elevated levels of SRSF1, the degradation of *SRSF1* transcripts *via* RNA surveillance pathways, such as nonsense-mediated decay (NMD), is induced through the removal of introns (77, 138, 142). Thus, upon strongly elevated levels of freshly synthesized *SRSF1* mRNA, the negative feedback loop could induce a higher frequency in intron removal, possibly resulting in the observed strong downregulation in *SRSF1* mRNA transcripts at later time points. Furthermore, SRSF1 activity and subcellular localization is regulated *via* phosphorylation (77, 143). Only partially phosphorylated SRSF1 binds its RNA target sequences with high affinity, while unphosphorylated or fully phosphorylated SRSF1 only has low binding affinities (60). Thus, an interaction between IFN-I-signaling and proteins regulating SRSF1 phosphorylation or dephosphorylation, affecting SRSF1 activity and localization, cannot be excluded.

SRSF1 consists of two RRMs, providing the RNA-binding specificity, and a relatively short RS domain (54). The purine-rich pentamer GGAGA was identified as SRSF1 consensus motif *via in vivo* mapping (76, 77). Interestingly, while SRSF1 mainly binds in ESE regions, introns contain a high number of potential binding sites (54). SRSF1 was previously shown to regulate multiple steps of HIV-1 RNA processing, including alternative splice site usage (78–80) and LTR promoter activity (83, 84), thus hinting at an important function of SRSF1 in HIV-1 RNA processing and replication. SRSF1 and the viral protein Tat competitively bind to a binding sequence within the hairpin structure of the TAR region on the LTR promoter (83). While increased levels of SRSF1 were shown to significantly impede Tat-activated LTR transcription, depleted levels resulted in enhanced LTR promoter activity (83, 84). In agreement with these data, we could show that siRNA-mediated knockdown of SRSF1 resulted in increased total viral mRNA transcript levels and thus HIV-1 LTR transcriptional activity (**Figure 8**), while SRSF1 overexpression led to a significant reduction in HIV-1 mRNA expression (**Figure 10**). Hence, a direct effect on SRSF1 on HIV-1 LTR transcription was suggested. Furthermore, alternative splice site usage was crucially affected by altered SRSF1 levels. Several SREs on the viral pre-mRNA were shown to be targeted by SRSF1, which are ESE M1/M2 (79), GAR ESE (78), and ESE3 (80), thereby enhancing the recognition of the proximal splice sites. Both overexpression and knockdown of SRSF1 led to significant alterations in the ratio of multiply spliced (spliced from SD4 to SA7) to unspliced (intron 1 containing) mRNAs (**Figures 7G** and **9G**). Overexpression of SRSF1 resulted in decreased levels of unspliced mRNAs but unaltered transcript levels of multiply spliced mRNAs, while knockdown shifted the ratio towards unspliced mRNAs. These findings were in agreement with SRSF1 enhancing splice site usage when binding SREs from an exonic position (61–63). Binding of SRSF1 to ESE3, which is located downstream of SA7 (80, 81), promotes the recruitment of U2AF65 to the SA, thus increasing splicing frequency at SA7 (81). The observed decrease in multiply spliced mRNAs upon SRSF1 knockdown might thus be explained through fewer splicing events at SA7 due to reduced SRSF1-mediated stabilization of U2 snRNP binding to the SA. Unaltered levels of multiply spliced mRNAs upon overexpression of SRSF1 were in accordance with a previous observation, showing that high levels of SRSF1 did not result in increased splicing frequency at SA7, possibly due to low binding affinity (81).

Cross-exon interactions play a key role in the recognition of exon 2 and exon 3 and concomitantly in the formation of *vif* and *vpr* mRNA (95, 144). Binding of the U1 snRNP to the 5’-SD, which stabilizes the binding of U2 snRNP to the branch point sequence and initiates the formation of an exon recognition complex (145), activates the upstream 3’-SA. We found that knockdown of SRSF1 induced a strong decrease in exon 2 inclusion and *vif* mRNA expression (**Figures 7D, E**), thus suggesting a strong reduction in splicing frequency at SA1. Since SRSF1 overexpression resulted in strongly increased *vif* mRNA levels, but not exon 2 inclusion (**Figures 9D, E**), splicing frequency at SA1 was likely enhanced, while splicing at SD2 and most likely at the alternative splice site SD2b (114) was blocked. ESE M1/M2 is located upstream of SD2 and was shown to promote the recognition of exon 2 *via* enhanced cross-exon interactions upon binding of SRSF1 (79). Thus, increased levels of SRSF1 likely resulted in increased recognition of SD2, thereby enhancing cross-exon interactions between SD2 and SA1. Depleted levels of SRSF1 resulted in fewer binding to ESE M1/M2, thereby resulting in lower splicing frequency at SA1, which, in turn, leads to reduced levels of exon 2 including and *vif* mRNA transcripts. Furthermore, while knockdown of SRSF1 resulted in strongly reduced exon 3-containing and *vpr* mRNAs (**Figures 7D, E**), overexpression concomitantly induced *vpr* mRNA expression but reduced exon 3 inclusion (**Figures 9D, E**). Recognition of SD3 was likely increased upon elevated levels of SRSF1, resulting in enhanced cross-exon interactions and thus splicing frequency at SA2. Impaired binding of U1 snRNP to SD3 could presumably lead to reduced exon 3 spanning cross-exon interactions. However, no SREs targeted by SRSF1 have been identified so far in proximity of SD3. The suggested reduction in splicing frequency at SA1 and SA2 upon SRSF1 knockdown was further sustained by the reduction of other exon 2 (*tat2* and *nef3*)- and exon 3 (*tat3* and *env8*)-containing mRNA levels. Moreover, induced mRNA levels of exon 2- and exon 3-containing mRNAs (*vpr2*, *tat4*, *tat8*, and *nef5*) upon elevated levels of SRSF1 supported the assumed increase in splicing events at SA1 and SA2.

Increased levels of SRSF1 also induced the repression of *env1* and *nef2*, which are spliced from SD1 to SA4 (36). A direct involvement of the bidirectional GAR ESE, which is located downstream of SA5 and was shown to regulate splicing frequency at SA4 (78, 146), was suggested. Upon binding of SRSF1 to GAR ESE, the recruitment of U1 snRNP to SD4 is enhanced, which increases splicing events at the upstream SA4 and SA5 (78, 146). However, in this study, reduced splicing frequency at SA3 was observed upon elevated levels of SRSF1 (**Figure 9F**). This could possibly be explained through the high complexity and dynamic of the splicing process, which involves a large number of proteins and cellular splicing factors. Potential interactions with other factors might thus impede the stabilizing effect of SRSF1 on the binding of U1 snRNP to SD4.

Unbalanced splicing of HIV-1 mRNAs drastically impairs viral replication and infectivity. In particular, optimal Vif levels are essential for efficient viral replication in APOBEC3G-expressing cells (13, 112, 147). While low levels of Vif resulted in a complete failure in viral replication due to the inability to evade APOBEC3G-mediated effects (95), elevated levels of Vif strongly decreased viral infectivity through the inhibition of Gag processing at the nucleocapsid (112).

Although SRSF1 knockdown induced a strong reduction in *vif* mRNA expression, facilitated viral infectivity was observed (**Figure 8**). Most likely, the strong increase in all viral RNAs as determined by levels of exons 1 and 7 compensate for the reduced *vif* mRNA expression and prevails as the dominant phenotype. Drastically reduced viral infectivity was observed upon SRSF1 overexpression (**Figure 10**). In agreement with previous findings (112), strongly elevated levels of *vif* mRNA resulted in a significant decrease in viral infectivity due to modulation of the proteolytic Gag processing. A Vif-independent mechanism including a direct effect of SRSF1 or the interaction of other cellular factors, however, cannot be ruled out.

Virus production was marginally elevated upon depleted levels of SRSF1 (**Figures 8C, D**), which was in accordance with the increase in HIV-1 LTR promoter activity (**Figure 7B**) and Gag expression (148). Overexpression of SRSF1 led to strongly reduced viral copies in the cellular supernatant (**Figure 10D**), in accordance with the strong inhibition of LTR promoter activity (**Figure 9B**). In addition, further overexpression effects were described including reduced Tat, Gag, and Env levels as well as the aforementioned inhibition of Tat transactivation (148).

Knockdown of SRSF1 resulted in marginally increased HIV-1 LTR transcription and viral infectivity. Thus, the observed IFN-induced repression of SRSF1 does not seem to have an antiviral effect and might even be detrimental in the defense against HIV-1. In contrast, high SRSF1 levels drastically impaired HIV-1 LTR transcription and viral infectivity. Hence, elevated levels of SRSF1, which were observed at an early time point after IFN stimulation, might be part of the IFN-induced antiviral response in the early immune response to HIV-1.

Interestingly, a concomitant HIV-1 infection and IFN stimulation led to rapidly restored SRSF1 levels comparable to PBS-treated cells (**Figure 4E**). However, whether the virus-mediated alteration in *SRSF1* mRNA levels was due to sensing or a direct effect mediated by viral proteins remained unclear. At the chosen time point of IFN stimulation 16 h post-infection, viral integration is complete and viral mRNA expression is proceeding (96). To evaluate whether viral proteins might be involved in SRSF1 regulation, we performed a transient transfection analysis. Overexpression of Vpr resulted in increased *ISG15* mRNA expression (**Supplementary Figure 3A**), which was in agreement with previous studies showing that Vpr is involved in the majority of changes in gene expression after HIV-1 infection (149). An NL4-3 derivate lacking Vpr only induced a limited ISG15 response when compared to parental NL4-3 (**Supplementary Figure 3**). These findings strengthen the hypothesis of a direct interplay between HIV-1 accessory proteins and host cell proteins, resulting in enhanced SRSF1 expression levels after 24 and 48 h (**Figure 4E**). Furthermore, Tat and Rev (150, 151), as well as the accessory protein Nef, are among the early expressed HIV-1 proteins (152). Rev was previously shown to interact with SRSF1 *via* the splicing factor-associated protein p32 (153), which was suggested to ensure optimal stoichiometry of HIV-1 mRNA isoforms (154). Certain accessory proteins like Nef might, in addition to counteracting restriction factors such as tetherin (14, 155), also modulate signal transduction, autoregulation mechanisms, antigen presentation, or the expression of cell surface receptors [reviewed in (156–158)]. Since an autoregulatory feedback loop was shown to maintain homeostatic levels of SRSF1 (77, 138, 142), interaction of accessory proteins with this autoregulation might potentially explain the observed recovery in the mRNA expression kinetic (**Figure 4E**). Experimentally, it is challenging to distinguish between HIV-1-infected cells producing IFNα and bystander cells, which induce gene expression of HIV restriction factors prior to the infection, thus inhibiting pre-integration steps. Further experiments will be required to distinguish the effect of modulated SRSF1 levels between these two cell populations.

Aside from being a crucial regulator of several HIV-1 post-integration steps, SRSF1 has a much broader scope of action. Among a crucial role in cellular alternative splicing (143), SRSF1 regulates genome stability (159), translation (160), nuclear export (69), or the nonsense-mediated mRNA decay (NMD) pathway (161, 162). Loss of SRSF1 protein function resulted in G2 cell cycle arrest and induced apoptosis (163). Moreover, SRSF1 was defined as a proto-oncogene, since upregulation of SRSF1 favors the formation of a variety of cancers (133, 140, 141). Thus, the IFN-induced downregulation of *SRSF1* described in this manuscript might not only affect HIV-1 post-integration steps, but also a variety of other cellular functions. Restoring balanced levels of SRSF1 after IFN treatment is essential for normal cell physiology, as prolonged unbalanced levels might have detrimental consequences.

As key factor for efficient HIV-1 replication, alternative splicing has been investigated as a potential target for ART (148, 164). An interesting question that remains is whether drug targeting of SRSF1 could potentially result in viral inhibition. The drug IDC16 was shown to block the replication of CXCR4- and CCR5-tropic viruses, as well as clinical isolates *via* direct interaction with SRSF1 (165, 166). The indole derivative can significantly impair the ESE activity of SRSF1 through binding of the RS domain and subsequently preventing its phosphorylation (165–167). Potential side effects of the drug are currently reviewed in *in vivo* studies. Current antiviral therapy is based on the inhibition of viral proteins, such as protease, integrase, or reverse transcriptase (168, 169). However, long-term use as well as inconsistent intake of these drugs can result in the emergence of drug-resistant mutations and can cause severe side effects (169). Thus, targeting cellular factors crucially involved in the regulation of HIV-1 post-integration steps may offer new approaches for antiviral therapy. However, the use of such drugs should be approached cautiously, due to the large number of cellular processes that are regulated by these factors.

This study had several limitations. We only had limited access to samples, in particular to those obtained from acutely infected individuals from different Fiebig stages (I–V). Factors such as age and gender, but also comorbidity and medication, nutrition, as well as other unknown environmental influences might have an impact on human gene expression including *ISG15* and *SRSF1* (142, 170–172). However, these data were not collected during this study and therefore cannot be used for further subgroup analysis. Another limitation of this study is the fact that SRSF1 knockdown and overexpression were performed in HEK293T cells. Although these cells are well established in the field, they do not represent natural HIV target cells. An efficient SRSF1 knockdown proved to be difficult in the course of the study, since, on the one hand, a complete knockout is lethal, while a minor knockdown is counteracted by an autoregulatory mechanism. Due to the technical complexity of achieving efficient SRSF1 knockdown, the question of its downregulation in natural host cells cannot be answered in this study and requires further research.

The different IFNα subtypes have been shown to exert different biological activities (173). Consistent with the observation that the IFNα subtypes induce different sets of ISGs (26), the different subtypes also showed clear differences in the ability to downregulate *SRSF1* mRNA expression (**Figure 3B**). While several IFNα subtypes elicit an antiviral activity suppressing HIV-1 infection, IFNα14 was shown to be the most potent subtype against HIV-1 *ex vivo* and *in vivo* (26, 27). In contrast to IFNα14, the subtype IFNα2, which is the sole subtype in clinical use against HBV (28), elicited only weak anti-HIV-1 activity (26, 27). In an *in vivo* humanized mouse model, it was shown that combined treatment of ART and IFNα14 led to a more efficient suppression in HIV-1 plasma viral load than ART treatment alone (174, 175). While a clinical study to test a concomitant administration of ART and IFNα2 has been carried out (https://clinicaltrials.gov/ct2/show/results/NCT02227277), a benefit of subtype IFNα14 when compared to IFNα2 for a potential use in therapy might have a higher effectiveness. However, since IFNα14 induces stronger intrinsic and innate immune responses (27), the administration of IFNα14 could also potentially cause more severe side effects when compared to IFNα2.

As a quintessence of this study, the IFNα-induced upregulation of HIV host restriction factors has been shown to severely limit viral replication (26, 27). In particular, pre-treatment with IFNα renders the cell into an antiviral state and inhibits numerous viral pre-integration steps (176). However, the IFNα-mediated deregulation of SRSF1 might at least partially direct the fate of viral replication and compensate for the antiviral activity by increasing the HIV-1 LTR transcription and virus production.

## Materials and methods

### Cell culture, transient transfection, and treatment

HEK293T, TZM-bl, Vero, and RPE ISRE-Luc reporter cells were maintained in Dulbecco’s modified Eagle medium (Gibco) supplemented with 10% (v/v) heat-inactivated fetal calf serum (FCS) and 1% (v/v) penicillin–streptomycin (P/S, 10,000 U/ml, Gibco). Jurkat and THP-1 cells were maintained in Roswell Park Memorial Institute (RPMI) 1640 medium (Gibco) supplemented with 10% (Jurkat) or 20% (THP-1) (v/v) heat-inactivated fetal calf serum (FCS) and 1% (v/v) penicillin–streptomycin (10,000 U/ml, Gibco). THP-1 monocytes were treated with 100 nM 12-*O*-tetradecanoylphorbol-13-acetate (TPA) for 5 days to differentiate into macrophage-like cells. Differentiation was monitored *via* cell morphology and adhesion.

Transient transfection experiments were performed in six-well plates at 2.5 × 10^5^ HEK 293 T cells per well using TransIT^®^-LT1 transfection reagent (Mirus Bio LLC) according to the manufacturer’s instructions unless indicated otherwise.

IFNα subtypes were produced as previously described (27); IFNγ was purchased from PBL assay science (Piscataway). For the stimulation with IFN, 10 ng/ml of the respective IFN was added in fresh medium to the cells. The cells were then incubated at 37°C and 5% CO_2_ for the indicated amount of time before being harvested. Ruxolitinib (*In vivo*gen) was resuspended in DMSO and further diluted in aqueous buffer. THP-1 cells were treated with either 1 µM Ruxolitinib or an equal volume of DMSO 1 h before infection. For each step involving media exchange, fresh Ruxolitinib was added.

The APOBEC3G-expressing HEK293T cells were previously stably transfected using the Sleeping Beauty system (177) and sorted in regard to their APOBEC3G expression.

### RNA isolation, quantitative, and semi-quantitative RT-PCR

The cells were harvested, and total RNA was isolated using the RNeasy Mini Kit (Qiagen) according to the manufacturer’s instructions. RNA concentration and quality were monitored *via* photometric measurement using NanoDrop2000c (Thermo Scientific). For reverse transcription (RT), 1 µg of RNA was digested with 2 U of DNase I (NEB). After heat inactivation of the DNase at 70°C for 5 min, cDNA synthesis for infection experiments was performed for 60 min at 50°C and 15 min at 72°C using 200 U SuperScript III Reverse Transcriptase (Invitrogen), 40 U RNase Inhibitor Human Placenta (NEB), 50 pmol Oligo d(T)23 (NEB), and 10 pmol Deoxynucleotide Triphosphate Mix (Promega). For all other experiments, cDNA synthesis was performed for 60 min at 42°C and 5 min at 80°C using the ProtoScript II First Strand cDNA synthesis kit (NEB) according to the manufacturer’s instructions. Quantitative RT-PCR analysis was performed using Luna^®^ Universal qPCR Master Mix (NEB) and Rotor-Gene Q (Qiagen). Primers used for qPCR are listed in **Supplementary Table 2**. ACTB or GAPDH was used as loading control for normalization. For qualitative analysis of HIV-1 mRNAs, PCR was performed using GoTaq G2 DNA Polymerase (Promega) according to the manufacturer’s instructions. PCR products were separated on non-denaturing polyacrylamide gels (12%), stained with Midori green Advanced DNA stain (Nippon Genetics), and visualized with ADVANCED Fluorescence and ECL Imager (Intas Science Imaging).

Plasma HIV-1 RNA level was quantified using the RealTime HIV-1 m2000 test system (Abbott) according to manufacturer’s instructions.

### IFN activity assay in the RPE ISRE-luc reporter cell line

A reporter cell line of human retinal pigment epithelial (RPE) cells, stably transfected with a plasmid containing the firefly luciferase reporter gene under the control of the IFN-stimulated response element (ISRE), was used to determine the activity of the different IFNα subtypes (27). Cells were seeded at 1.5 × 10^5^ cells per well in 12-well plates and incubated overnight. The next day, cells were stimulated with 10 ng/ml of the respective IFNα subtype for 5 h. Cells were then lysed with Passive lysis buffer (Promega) and frozen at −80°C overnight. After thawing, lysates were spun down and transferred to a white F96 Microwell plate (Nunc) before adding firefly luciferase substrate. Luminescent signal was measured using the GloMax^®^ Multi Detection System (Promega).

### Preparation of virus stocks and infection

For the preparation of virus stocks, 6.5 × 10^6^ HEK293T cells were seeded in T175 flasks coated with 0.1% gelatin solution. The next day, cells were transiently transfected with 19 μg of pNL4-3 or the respective proviral DNA using TransIT^®^-LT1 transfection reagent (Mirus Bio LLC) according to the manufacturer’s instructions. After 24 h, the cells were supplemented with Iscove’s Modified Dulbecco’s Medium [IMDM, 10% (v/v) FCS, and 1% (v/v) P/S] and incubated again overnight. The virus-containing supernatant was then purified by filtration through 0.30-μm MACS SmartStrainers (Miltenyi Biotec), aliquoted, and stored at −80°C. Differentiated THP-1 cells and Jurkat cells were infected with the R5-tropic NL4-3 (AD8) (1 MOI) or the dual tropic NL4-3 PI952 (1 MOI), respectively, with a spin inoculation for 2 h at 1,500 × *g*. Sixteen hours post-infection, indicated treatments were carried out.

### Protein isolation and Western blot

For protein isolation, cells were lysed with RIPA buffer [25 mM Tris-HCl (pH 7.6), 150 mM NaCl, 1% NP-40, 1% sodium deoxycholate, 0.1% SDS, and protease inhibitor cocktail (Roche)]. The lysates were subjected to SDS-PAGE under denaturing conditions in 12% polyacrylamide gels using the Bio-Rad Mini PROTEAN electrophoresis system (Bio-Rad). Gels were run for 90 min at 120 V in TGS running buffer [25 mM Tris, 192 mM glycine, and 0.1% SDS (v/v)]. NC membrane (pore size, 0.45 mm) was used for protein transfer using the Bio-Rad Mini PROTEAN blotting system (Bio-Rad). Proteins were transferred for 1 h at 300 mA in transfer buffer [25 mM Tris, 192 mM glycine, and 20% MeOH (v/v)]. The membrane was blocked in TBS-T [20 mM Tris-HCl, 150 mM NaCl, and 0.1% Tween-20 (v/v) (pH 7.5)] with 5% nonfat dry milk for 1 h at RT and then incubated overnight at 4°C with the primary antibody in TBS-T including 0.5% nonfat dry milk. The membrane was washed three times for 10 min in TBS-T. The horseradish peroxidase (HRP)-conjugated secondary antibody was added in TBS-T including 0.5% nonfat dry milk and incubated for 1 h at RT. The membrane was washed five times for 12 min with TBS-T before ECL chemiluminescent detection reagent (Amersham) was added and readout was performed with ADVANCED Fluorescence and ECL Imager (Intas Science Imaging). The following primary antibody was used: Mouse antibody specific for SRSF1 (32–4500) from Invitrogen. The following HRP-conjugated secondary antibody was used: anti-mouse HRP conjugate (315-035-048) from Jackson ImmunoResearch Laboratories Inc.

### p24-CA ELISA

For the quantification of HIV-1 p24-CA, a twin-site sandwich ELISA was performed as previously described (95). Briefly, Immuno 96 MicroWell plates (Nunc) were coated with α-p24 polyclonal antibody (7.5 µg/ml of D7320, Aalto Bio Reagents) in bicarbonate coating buffer (NaHCO_3_, 100 mM, pH 8.5) overnight at room temperature. The plates were washed with TBS and blocked with 2% non-fat dry milk powder in TBS for 1 h at room temperature. EMPIGEN twitterionic detergent (Sigma) was added to the samples for inactivation of HIV-1 and incubated for 30 min at 56°C. Capturing of p24 and subsequent washing were carried out according to the manufacturer’s instructions (Aalto Bio Reagents). An alkaline phosphatase-conjugated α-p24 monoclonal antibody (BC1071 AP, Aalto Bio Reagents) was used for quantification of p24. Readout was performed with the Spark^®^ Microplate Reader (Tecan). Recombinant p24 was used to establish a p24 calibration curve.

### TZM-bl Luc assay and X-Gal staining

A total of 4,000 TZM-bl cells were seeded per well in 96-well plates and incubated overnight. One hundred microliters of the virus-containing supernatant was added to the cells, and the plates were incubated for 48 h. For the luciferase assay, 50 µl of lysis juice (p.j.k.) was added after washing the plates with PBS, and the plates were shaken for 15 min at room temperature. Next, the plates were frozen for at least 1.5 h at −80°C before being thawed. Lysates were resuspended and transferred to a white F96 Microwell plate (Nunc) for luminescent readout. One hundred microliters of beetle juice (p.j.k.) was added per well, and luminescence was measured with the Spark^®^ Microplate Reader (Tecan) at an integration time of 2 s. For the X-Gal staining, cells were washed with PBS and fixed in 0.06% glutaraldehyde (Sigma) and 0.9% formaldehyde (Sigma) for 10 min at 4°C. Cells were washed twice with PBS and staining solution was added containing 400 mM K_3_[Fe(CN)_6_], 400 mM K_4_[Fe(CN)_6_], 100 mM MgCl_2_, and 20 mg/ml X-Gal. Cells were incubated overnight at 37°C and overlaid with 50% glycerol. Readout was performed optically with light microscopy.

### TZM-bl Luc assay of HEK293T APOBEC3G-expressing cells

A total of 4,000 TZM-bl cells were seeded per well in 96-well plates and incubated overnight. The virus-containing supernatant was serially diluted 1:3 in culture medium and 200 µl of the diluted viral supernatant was added to the cells. Forty-eight post-infection, cells were washed with PBS and lysed using 120 µl of lysis juice (p.j.k.). Plates were incubated for 15 min before a freeze-and-thaw cycle was applied, to ensure complete lysis. Thirty microliters of homogenized lysates were transferred to a white F96 Microwell plate (Nunc) for luminescent readout. One hundred twenty microliters of beetle juice (p.j.k.) was added per well and luminescence was measured using the GloMax Discover (Promega) with an integration time of 5 s with one reading per well.

### siRNA-based knockdown

If not indicated otherwise, HEK293T cells were transiently transfected with the indicated siRNA at a final concentration of 8 nM using Lipofectamine 2000 (Thermo Scientific) according to the manufacturer’s instructions. A further transfection was performed at a final concentration of 12.8 nM of the indicated siRNA to evaluate SRSF1 knockdown efficiency. The following siRNAs were used in this study: Silencer Select Negative Control #2 siRNA (Thermo Scientific) for the negative control siRNA and s12727 (Thermo Scientific) for SRSF1-specific siRNA.

### 4sU tagging

Differentiated THP-1 cells were treated for 30 min with 4sU (Sigma-Aldrich) at a final concentration of 500 µM for metabolic labeling of newly transcribed RNA following treatment with IFNα14 for the indicated amount of time. Labeling, purification, and separation of freshly transcribed RNA was carried out as described elsewhere (110). Newly transcribed RNA concentration and quality was measured using NanoDrop2000c (Thermo Scientific).

### PBMC isolation

PBMCs were isolated from whole blood samples by Ficoll density gradient centrifugation using LeucoSEP tubes (Greiner Bio-One) as described previously (178). RNA of isolated PBMCs was harvested as described above. This study has been approved by the Ethics Committee of the Medical Faculty of the University of Duisburg-Essen (14-6155-BO, 16-7016-BO, and 19-8909-BO). Consent form was not obtained since the data were analyzed anonymously.

### Statistical analysis

Differences between two groups were analyzed by unpaired two-tailed Student’s or Welch’s *t*-test. Multiple group analyses were performed using one- or two-way ANOVA followed by Bonferroni, Dunnett’s, or Tukey’s *post-hoc* test. Mixed models followed by Dunnett’s *post-hoc* tests were used for time-series analysis of multiple groups. A Kruskal–Wallis test with the Dunn’s *post-hoc* multiple comparisons test was applied to compare mRNA levels in PBMCs from acutely and chronically HIV-1-infected patients as well as from healthy donors due to violation of the assumptions for a parametric test. If not indicated differently, all experiments were repeated in three independent replicates. Asterisks indicated *p*-values as *p < 0.05, **p < 0.01, ***p < 0.005, and ****p < 0.0001.

## Data availability statement

Next-generation sequencing data were deposited in the NCBI Sequence Archive Bioproject with accession PRJNA422935. Further inquiries can be directed to the corresponding author.

## Ethics statement

The studies involving human participants were reviewed and approved by the Ethics Committee of the Medical Faculty of the University of Duisburg-Essen (14-6155-BO, 16-7016-BO, 19-8909-BO). Written informed consent for participation was not required for this study in accordance with the national legislation and the institutional requirements.

## Author contributions

HS: data curation, formal analysis, funding acquisition, investigation, methodology, project administration, visualization, writing-ODP, writing-RE. FR: investigation, writing-RE. AW: methodology, visualization. DH: investigation. BB: investigation. CE: investigation, resources, writing-RE. MS: data curation, investigation. JS: investigation, writing-RE. ZK: investigation. YB: investigation, resources. RS: investigation, resources. SE: resources. KS: funding acquisition, investigation, project administration, resources, writing-RE. UD: conceptualisation, funding acquisition, project administration, resources, supervision, writing-RE. MW: conceptualisation, funding acquisition, methodology, project administration, resources, supervision, visualization writing-ODP, writing-RE. All authors contributed to the article and approved the submitted version.

## Funding

These studies were funded by the DFG (WI 5086/1-1; SU1030/1-2), the Jürgen-Manchot-Stiftung (HS and MW), the Hessian Ministry of Higher Education, Research and the Arts (TheraNova, MW), and the Medical Faculty of the University of Duisburg-Essen (HS and KS). The authors thank the Jürgen-Manchot-Stiftung for the doctoral fellowship of Helene Sertznig.

## Acknowledgments

We thank Christiane Pallas for excellent technical assistance. We thank Heiner Schaal for providing plasmid DNA and Mirko Trilling for fruitful discussions. The following reagents were obtained through the AIDS Research and Reference Reagent Program, Division of AIDS, NIAID, NIH: TZM-bl cells from Dr. John C. Kappes and Dr. Xiaoyun Wu. pEGFP-SF2 (179) was a gift from Tom Misteli (Addgene plasmid # 17990; http://n2t.net/addgene:17990; RRID: Addgene_17990). pcDNA-FLAG-SF2 was a gift from Honglin Chen (Addgene plasmid # 99021; http://n2t.net/addgene:99021; RRID: Addgene_99021) (117).

## Conflict of interest

The authors declare that the research was conducted in the absence of any commercial or financial relationships that could be construed as a potential conflict of interest.

## Publisher’s note

All claims expressed in this article are solely those of the authors and do not necessarily represent those of their affiliated organizations, or those of the publisher, the editors and the reviewers. Any product that may be evaluated in this article, or claim that may be made by its manufacturer, is not guaranteed or endorsed by the publisher.
