# Supplementary material for: SRSF1 acts as an IFN-I-regulated cellular dependency factor decisively affecting HIV-1 post-integration steps
Source: Front Immunol. 2022 Nov 15;13:935800. doi: 10.3389/fimmu.2022.935800 (PMC9706209; doi:10.3389/fimmu.2022.935800)
Supplement: Supplementary file 1 [file DataSheet_1.pdf]

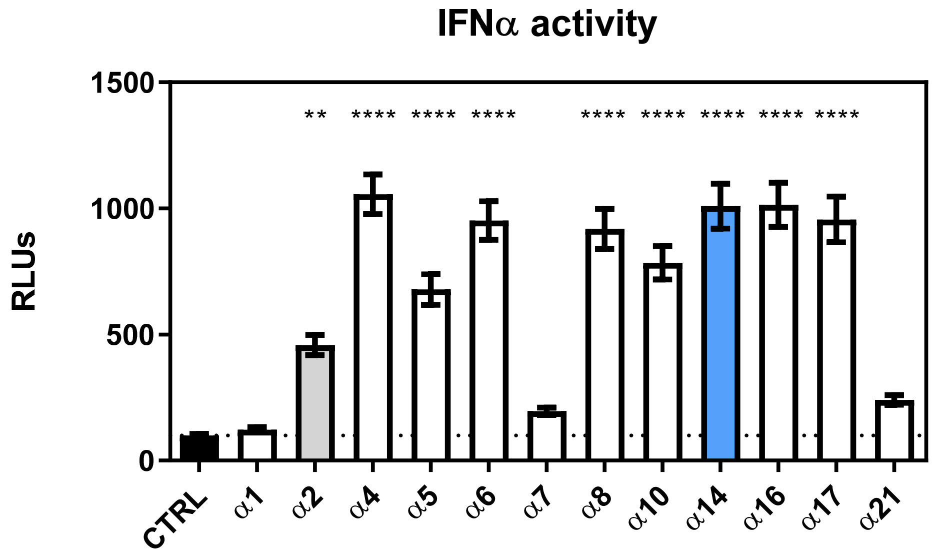

Supplementary Figure 1: Interferon alpha subtypes differ in their ability to activate ISRE. A luciferase reporter cell line harboring the firefly luciferase gene under the control of the ISRE promoter was stimulated with the indicated IFNα subtype at a concentration of 10 ng/ml. After 5 h, cells were harvested and luminescent signal measured after addition of firefly luciferase substrate. Statistical significance was determined by one-way ANOVA with Dunnett's *post hoc* test (* $p<0.05$, ** $p<0.01$, *** $p<0.001$, **** $p<0.0001$).

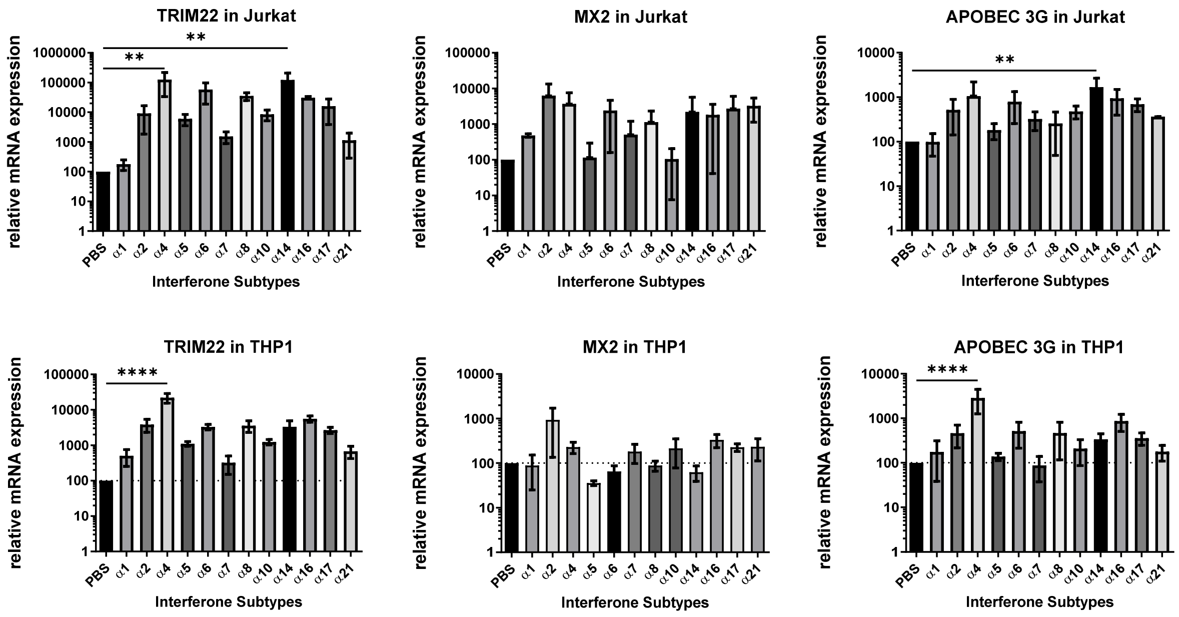

Supplementary Figure 2: Interferon alpha subtypes differ in their ability to stimulate expression of host restriction factors. Relative expression patterns of **(A)** TRIM22, **(B)** MX2 and **(C)** APOBEC 3G in Jurkat (upper panel) and THP-1 (lower panel) relative to PBS-treated cells. RT-qPCR data was normalized to relative beta actin expression. Experiments were performed in triplicates except for THP-1 IFNα21 (n=2) for all ISGs and IFNα7 (n=2) for Mx2 and for Jurkat IFNα8 (n=2) for all ISGs, IFNα1 (n=2) for Mx2 and IFNα21 (n=2) for APOBEC3G. Significance levels were determined using a one-way ANOVA followed by Dunnett's *post hoc* test and are given as follows: *p<0.05, **p<0.01, ***p<0.001.

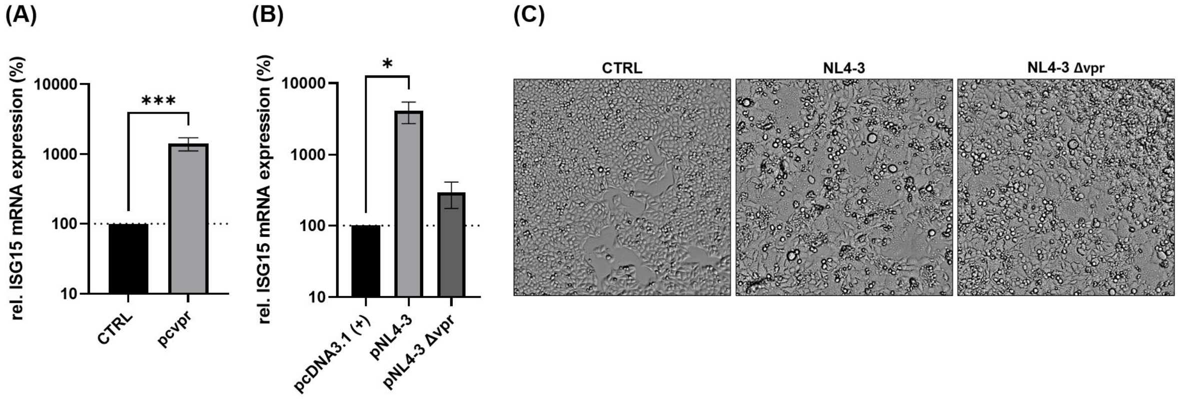

Supplementary Figure 3 - The HIV-1 accessory protein Vpr induces strong ISG15 expression. HEK293T cells were transfected with **(A)** subgenomic expression plasmid encoding the HIV-1 accessory protein Vpr (unpaired, two-sided Welch's t-test, *** p<0.001) or **(B)** with the full length HIV-1 proviral plasmid DNA clone (NL4-3) or the Vpr deficient derivate (NL4-3 Δvpr). Cells were rinsed with PBS 48 h (A) or 72 h (B) post transfection and subjected to RNA isolation. RT-qPCR was performed to evaluate ISG15 expression levels. Statistical significance was determined by one-way ANOVA with Dunnett's post hoc test (* p<0.05) **(C)** TZM-bl cells were seeded into 96-well plates and infected using the previously harvested supernatant from (B). 48 h post infection syncytia formation was qualitatively analyzed to monitor the production of infectious HIV-1.

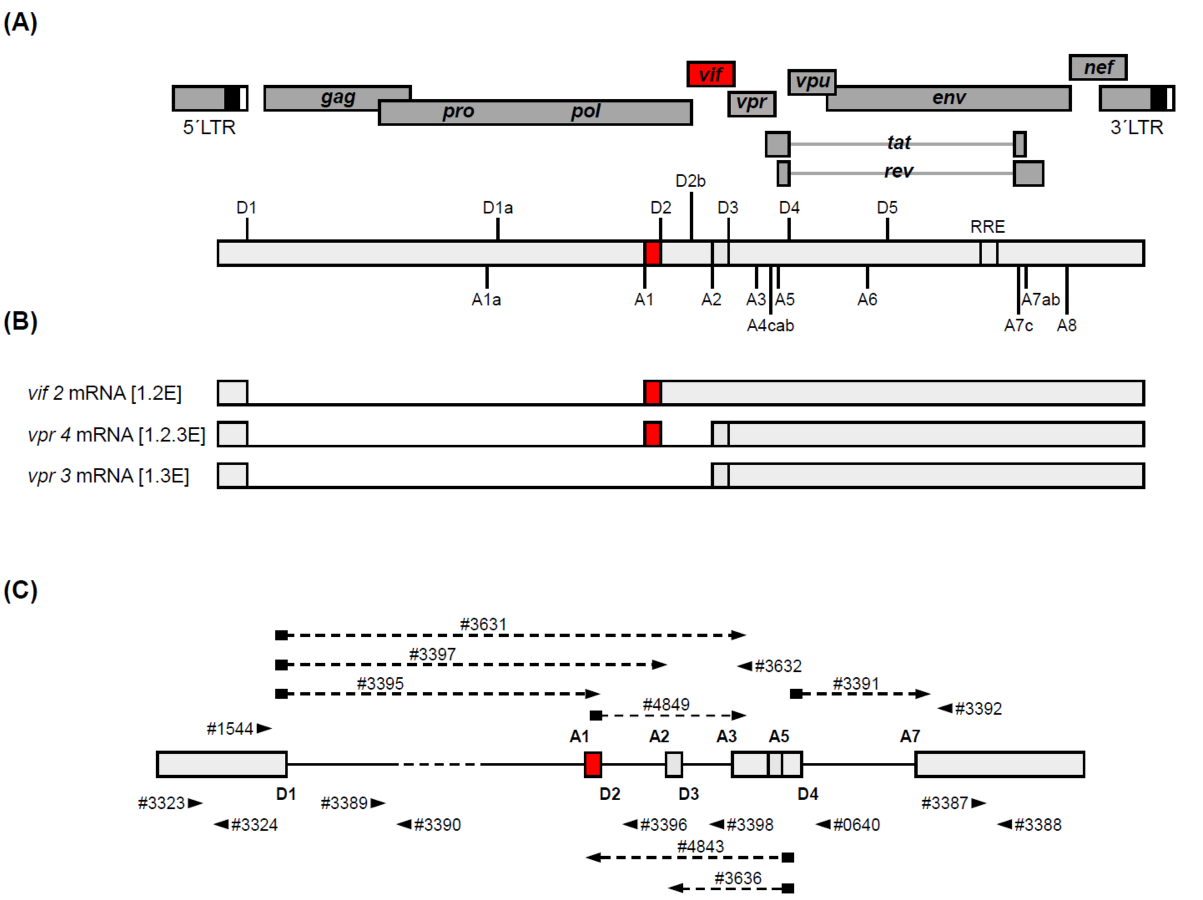

Supplementary Figure 4: HIV-1 NL4-3 genome. a) HIV-1 genome with open reading frames (ORFs) and long terminal repeats (LTRs). 5'- and 3'-splice sites are indicated as well as the Rev response element (RRE). *Vif* Exon and ORF is highlighted in red. **b)** *Vif* and *Vpr* mRNs are spliced from 5'-ss D1 to 3'-ss A1 and 5'-ss D1 to 3'-ss A2 respectively, harboring the non-coding leader Exons 2 and 3. AUG-containing Introns 2 and 3 are contained respectively. **c)** Binding sites of primers for RT-qPCR and RT-PCR. Grey boxes indicate Exons, while straight lines indicate Introns. Black arrowheads indicate primers. Primers with black rectangle and black arrowhead connected via dashed line indicate Exon-junction primers.

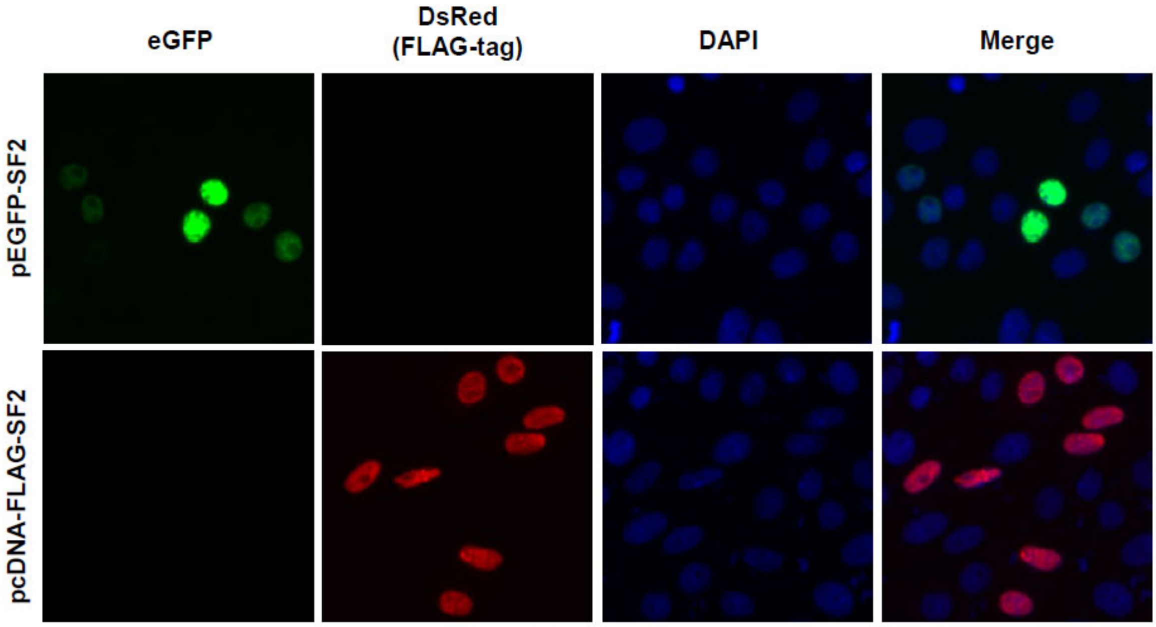

Supplementary Figure 5 - Immunofluorescence staining confirms expression and nuclear localization of exogenously transfected SRSF1. Vero cells were transiently transfected with pEGFP-SF2 (Phair and Misteli, 2000) or pcDNA-FLAG-SF2 (Huang et al., 2017). Cells were fixed 24 h post transfection and nuclei were stained using DAPI. Immunofluorescence microscopy of FLAG-tagged SRSF1 or EGFP-SRSF1 fusion proteins were performed to confirm expression and nuclear localization of exogenously transfected SRSF1.
