# Supplementary material for: SRSF1 acts as an IFN-I-regulated cellular dependency factor decisively affecting HIV-1 post-integration steps
Source: Front Immunol. 2022 Nov 15;13:935800. doi: 10.3389/fimmu.2022.935800 (PMC9706209; doi:10.3389/fimmu.2022.935800)
Supplement: Supplementary file 3 [file Table_2.docx]

## **Supplementary Tables**

**Supplementary Table 2:** Primers used for RT-PCR and RT-qPCR. Transcript specific primer pairs were used to quantitatively determine changes in the viral splicing pattern via RT-qPCR.

| Primer | Primer Sequence (5’-3’) | Target |
| --- | --- | --- |
| MW_1001  MW_1002  MW_1003  MW_1004  MW_1005  MW_1006  MW_1007  MW_1008  MW_1009  MW_1010  MW_1055  MW_1056  MW_3380  MW_3384  MW_3323  MW_3324  MW_3387  MW_3388  MW_3389  MW_3390  MW_3391  MW_3392  MW_3395  MW_3396  MW_3397  MW_3398  MW_3381  MW_3382  MW_3393  MW_3385  MW_3386 | CATCGAGCAC GGCATCGTCA  TAGCACAGCC TGGATAGCAA C  TGCACCACCA ACTGCTTA  GGATGCAGGG ATGATGTTC  GAGAGGCAGC GAACTCATCT  AGGGACACCT GGAATTCGTT  TTTGTATCGG CCTGTGTGAA TG  AAGCATGGCT GGGACATCA  GAGATGGCAC TGGTGTCGTG  TGCGACTCCT GCTGTTGCTT C  ACTCCGTGAAGTCTAGGGACA  TGTCACAGAGCCGAATACCAG  CAATACTACT TCTTGTGGGT TGG  CTTGAAAGCG AAAGTAAAGC  CTGAGCCTGG GAGCTCTCTG GC  GGGATCTCTA GTTACCAGAG  TTGCTCAATG CCACAGCCAT  TTTGACCACT TGCCACCCAT  TTCTTCAGAG CAGACCAGAG C  GCTGCCAAAG AGTGATCTGA  TCTATCAAAG CAACCCACCT C  CGTCCCAGAT AAGTGCTAAG G  GGCGACTGGG ACAGCA  CCTGTCTACT TGCCACAC  CGGCGACTGA ATCTGCTAT  CCTAACACTA GGCAAAGGTG  CGGCGACTGA ATTGGGTGT  TGGATGCTTC CAGGGCTC  CCGCTTCTTC CTTGTTATGT C  CCGCTTCTTC CTTTCCAGAG G  ACCCAATTCT TTCCAGAGG | ACTB fwd  ACTB rev  GAPDH fwd  GAPDH rev  ISG15 fwd  ISG15 rev  IRF1 fwd  IRF1 rev  SRSF1 fwd  SRSF1 rev  IFITM1 fwd  IFITM1 rev  HIV-1 4kb mRNA class  HIV-1 2kb-, 4kb-, tat mRNA class  HIV-1 exon1 fwd  HIV-1 exon1 rev  HIV-1 exon7 fwd  HIV-1 exon7 rev  HIV-1 unspliced mRNA fwd  HIV-1 unspliced mRNA rev  HIV-1 multiply spliced mRNA fwd  HIV-1 2kb mRNA class  HIV-1 multiply spliced mRNA rev  HIV-1 vif mRNA fwd  HIV-1 tat2 mRNA fwd  HIV-1 exon2 incl. mRNA fwd  HIV-1 vif mRNA rev  HIV-1 vpr mRNA fwd  HIV-1 tat3 mRNA fwd  HIV-1 exon3 incl. mRNA fwd  HIV-1 vpr mRNA rev  HIV-1 tat1 mRNA fwd  HIV-1 tat1 mRNA rev  HIV-1 tat3 mRNA rev  HIV-1 tat mRNA class  HIV-1 exon3 incl. mRNA rev  HIV-1 exon2 incl. mRNA rev  HIV-1 tat2 mRNA rev |
